# A conserved partner-switching system controls terminal differentiation in multicellular cyanobacteria

**DOI:** 10.1128/mbio.01685-26

**Published:** 2026-09-03

**Authors:** Stéphanie Champ, Eva Furet, Frédérique Pompeo, Xiaomei Xu, Sylvain Lemeille, Marine Novelli, Zorah Dermoun, Matthieu Bergé, Emmanuel Talla, Amel Latifi

**Affiliations:** 1 Aix Marseille Univ, CNRS, Laboratoire de Chimie Bactérienne 128791, Marseille, France; 2 Department of Pathology and Immunology, University of Geneva 27212, Geneva, Switzerland; South China Sea Institute of Oceanology, Guangzhou, Guangdong, China

**Keywords:** gene expression, cyanobacteria, transcriptional regulation, cell differentiation, genomics

## Abstract

Canonical partner-switching systems (PSSs) regulate sigma factor activity through reversible phosphorylation, but their established roles have been largely limited to stress responses and sporulation in Firmicutes. Whether this regulatory mechanism also controls developmental cell fate decisions in other bacterial phyla has remained unknown. Here, we identify a canonical PSS that governs heterocyst differentiation in the multicellular cyanobacterium *Anabaena* sp. PCC 7120. This system comprises the anti-sigma factor All2284 (NfsS) and the anti-anti-sigma factor All2283 (NfsR). Structural predictions and biochemical assays showed that NfsS phosphorylates NfsR on a conserved serine residue, whereas bacterial two-hybrid and co-purification assays demonstrated that NfsS binds the developmental sigma factors SigC and SigE. Deletion of *nfsR* abolished heterocyst formation and diazotrophic growth, and transcriptomic analysis revealed broad failure to induce late heterocyst genes, including nitrogen fixation functions such as *nifHDK* and *fdxH*. Phylum-wide comparative genomics further showed that PSS genes and putative functional clusters are strongly enriched in filamentous and heterocyst-forming taxa, indicating an association between the expansion of these signaling modules and the emergence of multicellularity and developmental specialization. Together, these findings establish a PSS as a direct regulator of terminal cell differentiation in a gram-negative bacterium and reveal partner switching as a conserved regulatory principle linking environmental signaling to developmental fate in a major bacterial phylum.

**IMPORTANCE:**

While partner-switching systems are classically associated with stress responses and sporulation control in Firmicutes, whether this regulatory logic governs developmental decisions in other bacterial phyla has remained unknown. Here, we establish that a related partner-switching mechanism operates in a distinct bacterial lineage, the cyanobacteria, where it controls a major developmental transition involving terminal cell differentiation. Specifically, we show that a phosphorylation-dependent checkpoint involving the anti-sigma factor NfsS and the anti-anti-sigma factor NfsR directly regulates heterocyst formation. Disruption of this switch abolishes cell differentiation and diazotrophic growth, revealing that this system is an obligate gatekeeper for terminal differentiation. Conceptually, these findings substantially extend the known functional repertoire of partner-switching circuits: rather than controlling stress adaptation or spore dormancy, this module has been co-opted to govern a complex, multicellular developmental program in an organism that underpins global carbon and nitrogen cycles. This work, therefore, establishes a new paradigm for phosphorylation-based control of developmental sigma factors and provides a tractable model for dissecting how conserved signaling modules are rewired to drive lineage-specific innovations across the bacterial domain.

## INTRODUCTION

The ability of bacteria to thrive and adapt relies on the fine-tuned control of gene transcription, which allows them to rapidly adjust gene expression in response to environmental or physiological signals. Central to this control are sigma (σ) factors, RNA polymerase subunits that direct promoter recognition and orchestrate the activation of specific regulons (1). Bacteria typically encode multiple sigma factors, including both housekeeping and alternative sigma factors, the latter allowing selective activation of genes involved in stress responses, developmental programs, or metabolic adaptations (2). Since sigma factors compete for the core RNA polymerase, their activity needs to be precisely controlled. One such mechanism is the partner switch system (PSS), a phosphorylation-driven regulatory circuit that controls gene expression by sequestering or releasing a sigma factor. Its four core components are the sigma factor, its inhibitory anti-sigma factor (also a kinase), the anti-anti-sigma factor, and a phosphatase (3).

**Fig 1:**
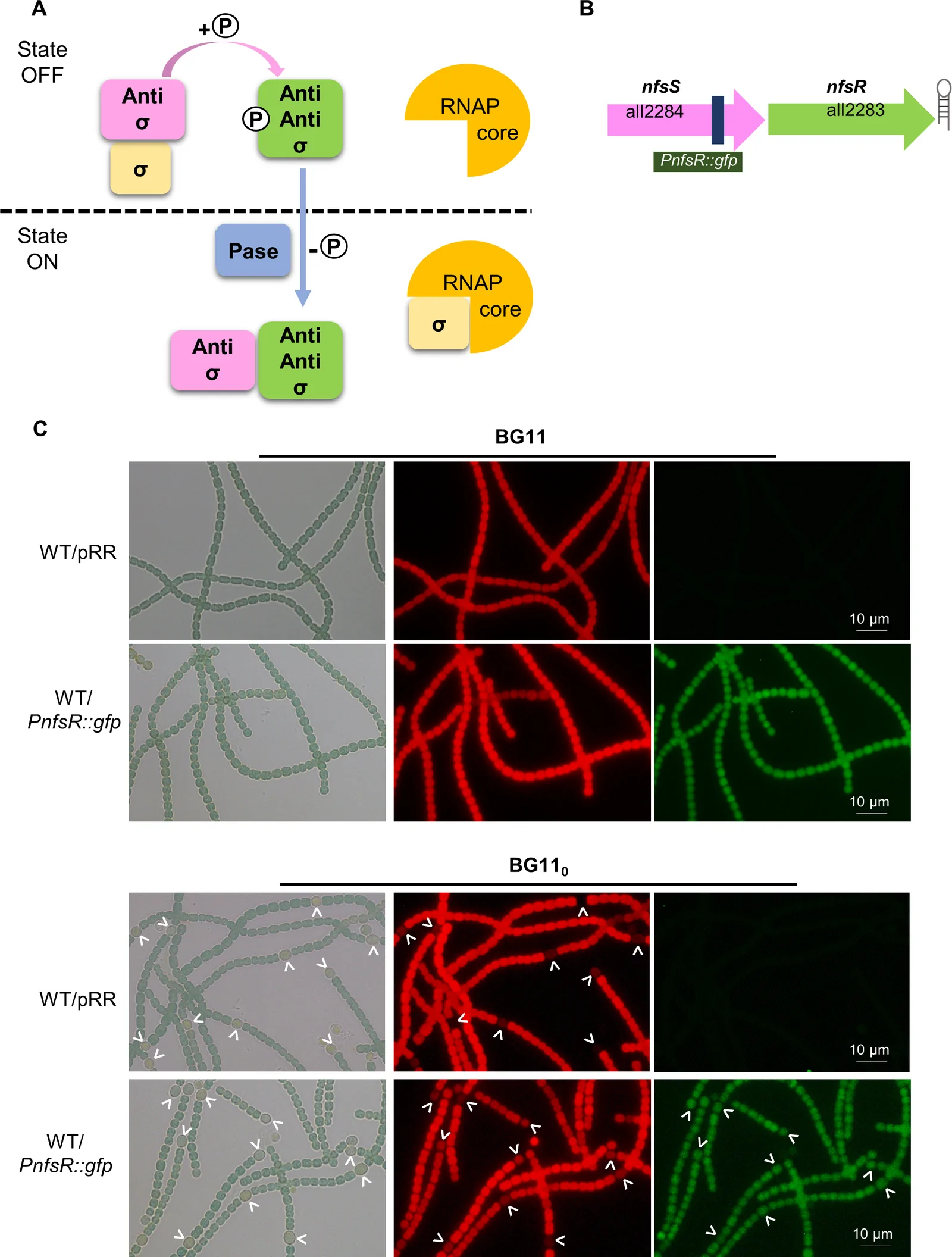
The Nfs PSS in *Anabaena*. (**A**) Schematic representation of the function of a typical PSS. State OFF (absence of signal): transcription off. State ON (presence of signal): transcription on. Abbreviations: RNAP core, RNA polymerase core enzyme; *σ* (s), sigma subunit; Anti*-*σ (Anti s), anti-sigma factor; Anti-ant*i-*σ (Anti-anti-s), anti-anti-sigma factor; Pase, phosphatase; and P, phosphorylated state. In the absence of a signal, the anti-sigma factor sequesters the sigma factor (σ) and phosphorylates the anti-anti-sigma factor. In the presence of a signal, the phosphatase dephosphorylates the anti-anti-sigma factor, which then binds the anti-sigma factor, releasing the sigma factor to activate transcription. (**B**) Chromosomal organization of the *nfs* PSS-gene cluster in the genome of *Anabaena*. Gene colors correspond to those of panel A (pink, anti-sigma; green, anti-anti-sigma factor). The dark blue box indicates NtcA-binding sites identified by ChIP-seq analysis. The hairpin scheme indicates the predicted transcriptional terminator. The dark green box indicates the *nfsR* promoter region used in the *PnfsR::gfp* fusion. (**C**) Expression profile of the *PnfsR::gfp* fusion. Microscopic bright-field images (left), auto-fluorescence images (middle), and GFP fluorescence (right) of the indicated *Anabaena* strains grown in BG11 (top) or 24 h after nitrogen stepdown (bottom). White arrows point to heterocysts.

By default, the anti-sigma factor binds and inactivates the sigma factor while also phosphorylating (and thus inactivating) the antagonist. A detected signal triggers the phosphatase to dephosphorylate the antagonist. Upon dephosphorylation, the antagonist then binds the anti-sigma factor, forcing it to release the sigma factor (Fig. 1A) (3).

Partner switch systems have been best characterized in *Bacillus subtilis*, where the general stress response is regulated by the SigB pathway. Under non-stress conditions, the anti-sigma factor RsbW inhibits SigB and phosphorylates the anti-sigma antagonist RsbV. In response to stress, RsbV is dephosphorylated upon the activation of the serine phosphatase RsbU, leading to stress-responsive gene activation (4). A similar system (SpoIIAA-SpoIIAB-SigF) regulates sporulation in Firmicutes (5), demonstrating the role of PSS in cell differentiation beyond stress response.

Numerous PSS regulating diverse cellular functions have been identified across both gram-positive and gram-negative bacteria, many of which exhibit unique features, including multidomain proteins and complex multipartner interactions, as well as various mechanisms that activate the phosphatase (3). For instance, in the unicellular cyanobacterium *Synechocystis* sp. PCC 6803, SigC availability is controlled by the Slr1861-Ssr1600 partner-switching module, in which the anti-sigma factor Slr1861 phosphorylates the anti-sigma antagonist Ssr1600, thereby adjusting RNA polymerase composition in response to environmental inorganic carbon levels (6). In the same organism, Ssr1600 is also targeted by PmgA, a partner-switching component related to anti-sigma factors, which contributes to high-light acclimation by phosphorylating Ssr1600 and thereby links this stress-response pathway to the control of SigC availability (7). More broadly, partner-switching-like systems illustrate the diversification of these regulatory modules, in which anti-sigma antagonist-like proteins can function as central regulatory outputs, while the downstream connection to transcription may involve sigma factors, remain indirect, or be unresolved. Examples include the RbaW-RbaV-RbaY system—which modulates gene transfer agent (RcGTA) production in *Rhodobacter capsulatus,* although direct interaction assays with candidate sigma factors did not reveal an RbaW-sigma factor interaction (8)—and the HmpU-HmpV-HmpW system—which controls hormogonium development in the filamentous cyanobacterium *Nostoc punctiforme* through an HmpV-dependent pathway whose downstream target remains undefined (9).

Alternatively, sigma factors can also be sequestered by proteins that are not part of the PSS family. One such instance is the extracytoplasmic sigma factor SigG, which is involved in cellular stress response in *N. punctiforme* (10). SigG was shown to interact *in vitro* with the anti-sigma factor SapG, a protein that shares no homology with PSS-related anti-sigma factors (10), indicating that non-PSS mechanisms can achieve analogous regulatory outcomes through structurally distinct components.

Cyanobacteria are the only oxygenic photosynthetic bacteria and exhibit a broad ecological distribution. They have evolved diverse strategies to adapt to fluctuating nutrient conditions (11). Their genomes encode a multitude of alternative sigma factors, particularly from group 2, which play crucial roles in modulating gene expression under varying environmental conditions (12, 13). In the filamentous cyanobacterium *Anabaena* (*Nostoc*) sp. PCC 7120, a subset of cells differentiates into heterocysts—specialized, terminal cells dedicated to atmospheric nitrogen fixation. This process requires coordinated repression of photosynthesis, induction of nitrogenase genes, remodeling of cell envelopes, and the establishment of intercellular communication within the filament (14). It is driven by a complex gene regulatory network involving global transcription factors (e.g., NtcA), developmental regulators (e.g., HetR), and cell-cell signaling molecules (14). Upon combined nitrogen starvation, NtcA interacts with 2-oxoglutarate (a key metabolic signal triggering differentiation (15)) and activates heterocyst development by directly or indirectly regulating the expression of multiple genes, including *hetR*, the master regulator of differentiation (16).

*Anabaena* encodes 12 sigma factors, three of which, SigC (All1692), SigE (Alr4249), and SigG (Alr3280), are induced under nitrogen-limiting conditions in a HetR-dependent manner (17). While SigC and SigE belong to group 2 and are homologous to those regulated by partner-switching systems, SigG is classified within the extracytoplasmic sigma factor family (group 3) (13, 18). In *N. punctiforme*, the *sigG* gene is co-localized with *sapG*, which encodes an anti-sigma factor that interacts with SigG (10). In *Anabaena*, the *sigG* gene is also co-localized with a neighboring gene whose predicted product shows homology to SapG from *N. punctiforme*, suggesting that SigG activity in *Anabaena* may be regulated by this adjacent putative anti-sigma factor through a conserved mechanism. HetZ, which shares structural homology with extracytoplasmic function sigma factors, was recently shown to act as a sigma factor controlling heterocyst differentiation and patterning by activating developmental genes downstream of initiation (19). PatU3, encoded in the same gene cluster as *hetZ*, inhibits HetZ activity and has been proposed as its cognate anti-sigma factor (19).

SigC and SigE have been shown to regulate numerous genes that are essential for heterocyst differentiation and function (17, 20, 21). Despite clear evidence that specific sigma factors are involved in heterocyst-specific gene expression, the upstream mechanisms that regulate the availability and activity of sigma factors during this process remain poorly understood.

Here, we report the discovery and functional analysis of a PSS in *Anabaena* that is essential for heterocyst differentiation. Our work reveals that this phosphorylation-dependent regulatory module interacts with sigma factors required for the transcriptional activation of heterocyst-specific genes. Through a combination of targeted genetic analysis, global RNA sequencing, *in vitro* biochemical assays, and comparative genomics, we demonstrate that this system functions as a developmental checkpoint that activates the heterocyst transcriptional program and has likely played a crucial role in the emergence and development of multicellularity in cyanobacteria. These findings uncover a previously unrecognized layer of transcriptional regulation in cyanobacteria and highlight the evolutionary versatility of partner switch mechanisms.

## RESULTS

To identify a PSS potentially involved in cell differentiation in *Anabaena*, we searched for proteins containing the core functional domains of known PSS factors (Table S1, sheets 1 and 2) (3). The anti-sigma factor family is characterized by the HATPase domain, a hallmark of histidine kinase-like ATPases (22). Anti-sigma antagonist (AAS) proteins typically contain a sulfate transporter anti-sigma factor antagonist (STAS) domain, which features a conserved serine residue that undergoes regulatory phosphorylation (23). Finally, PSS-associated phosphatases contain either a PP2C or a SpoIIE domain. The PP2C domain is a Mn²^+^/Mg²⁺-dependent phosphatase responsible for dephosphorylating phosphoserine/threonine residues (24). The SpoIIE domain, first identified in the *B. subtilis* SpoIIE protein, is involved in activating the cell-specific sigma factor σ^F^ (25).

Through our analysis, we identified 22 genes encoding putative homologs of PSS components. These fall into three functional classes: 5 anti-sigma factors, 7 anti-anti-sigma factors, and 10 phosphatases (Table S1, sheet 3). Among these, two putative PSS clusters, each containing genes in close chromosomal proximity, appear to encode complete PSS modules. Each module includes an anti-sigma factor, its antagonist, and a phosphatase (Table S1, sheet 3).

Of interest was a system consisting of all2283 (predicted anti-anti-sigma factor) and all2284 (anti-sigma factor) (Fig. 1B). Significantly, ChIP-seq demonstrated that NtcA, the global transcriptional regulator essential for nitrogen assimilation and heterocyst differentiation, binds directly to the upstream regulatory regions of all2283 (26). This observation suggests that NtcA controls the anti-anti-sigma-encoding gene, integrating it into the broader regulatory network that orchestrates heterocyst differentiation under nitrogen stress. We therefore focused our investigation on this candidate system to address three key questions: whether its genes are expressed under nitrogen-fixing conditions, whether they function together as a bona fide partner-switching module (i.e., via phosphorylation), and whether this module is required for proper heterocyst differentiation.

For clarity and consistency throughout this study, we designate the genes constituting this putative partner switch system as *nfs* genes, standing for nitrogen fixation switch. In this nomenclature, *nfsS* refers to the anti-sigma factor (sigma sequestrator, all2284), and *nfsR* to the anti-anti-sigma factor (sigma regulator, all2283).

### The *nfsR* gene is expressed in vegetative cells and heterocysts under nitrogen-fixing conditions

Since the anti-anti-sigma factor functions as the key regulator that promotes the release of the sigma factor, we first focused on characterizing its encoding gene, *nfsR* (all2283).

RNA sequencing analyses revealed that the transcription of *nfsR* is upregulated 2.9-fold 12 h after combined nitrogen deprivation (27). To characterize *nfsR* expression patterns, we generated a fusion of its promoter region with GFP. Under nitrogen-replete conditions, the fusion showed detectable GFP fluorescence (Fig. 1C; Fig. S1). When assayed 24 h after nitrogen step-down, fluorescence was observed in both vegetative cells and heterocysts (Fig. 1C; Fig. S1). The absence of a detectable increase in GFP signal between nitrogen-replete and nitrogen-starved conditions may be explained by the already high basal expression of the *nfsR* promoter, which could mask the 2.9-fold transcriptional induction observed by RNA-seq. Collectively, these findings confirm that *nfsR* is expressed under nitrogen-fixing conditions, prompting further investigation into its contributions to nitrogen fixation and heterocyst differentiation.

### NfsS phosphorylates NfsR on a serine residue conserved in PSS-type anti-anti sigma factors

In bacterial phosphorelay systems, genes encoding anti-sigma factors and their antagonists are often co-localized, a genomic arrangement that typically reflects functional coupling. The physical proximity of *nfsR* and *nfsS* suggests that they operate within the same regulatory module. This hypothesis is reinforced by both sequence and structural data. Multiple sequence alignments highlight conserved motifs: NfsR harbors a serine residue consistent with phosphorylation-dependent regulation, while NfsS features a conserved asparagine residue critical for kinase activity (Fig. S2).

**Fig 2:**
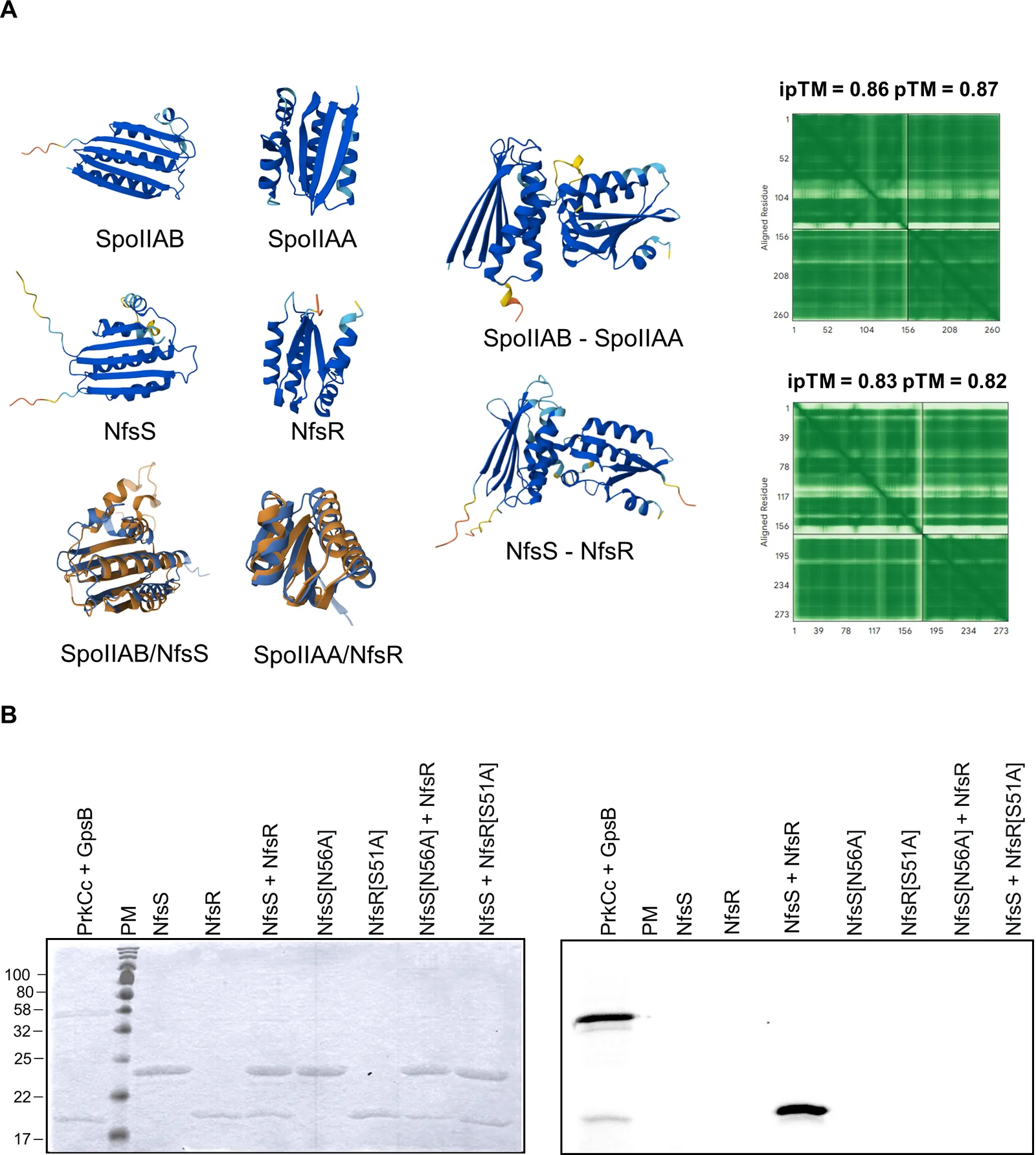
Structural predictions and *in vitro* validation of the NfsS-NfsR relationship. (**A**) AlphaFold-Multimer predictions of the *B. subtilis* SpoIIAB-SpoIIAA and *Anabaena* NfsS-NfsR systems. Left panel: structure models of the full-length proteins, colored by chain, and a superposition of the predicted structures for the two classes of factors (anti-sigma factors: SpoIIAB and NfsS; anti-sigma antagonists: SpoIIAA and NfsR). Right panel: for each system, the predicted complex structure is shown (cartoon, colored by chain) and, next to it, the corresponding all-against-all residue contact-probability heat map; darker green indicates higher confidence. pTM, predicted global template-modeling score; iPTM: interface-specific TM score, reflecting the reliability of the inter-chain contact prediction. (**B**) *In vitro* kinase assay of the anti-sigma factor NfsS. Left panel: Coomassie blue staining of SDS-PAGE gel. Right panel: autoradiography of the corresponding SDS-PAGE gel. The proteins used in each assay are indicated at the top of the figure. PrkC and GpsB were used as positive controls for the phosphorelay assay. The experiment was repeated three times, yielding similar results. The calculated molecular masses of His-NfsS and His-NfsR are approximately 21 and 15.1 kDa.

The structures of SpoIIAA (pdb_00001auz) (28) and SpoIIAB (as part of the SpoIIAA-SpoIIAB complex, pdb_00001til) (29) from *B. subtilis* have been experimentally resolved, providing well-characterized models of anti-anti-sigma and anti-sigma kinase folds, respectively. AlphaFold models indicate that NfsR adopts a SpoIIAA-like fold, typical of anti-anti-sigma, whereas NfsS closely resembles the anti-sigma kinase SpoIIAB from *B. subtilis* (Fig. 2A). Notably, AlphaFold also predicts a high-confidence interaction between the two proteins, supported by robust pTM and iPTM scores, predicting the formation of a stable complex (Fig. 2A). Taken together, their chromosomal colocation, conserved functional residues, structural homology, and predicted interaction strongly support the view that NfsR and NfsS constitute a cognate pair within a partner-switch regulatory system.

To test whether NfsS can directly phosphorylate NfsR, we performed *in vitro* phosphorylation assays using purified His-tagged recombinant proteins produced in *Escherichia coli* (Fig. S3A). Upon incubation with [γ-³³P]ATP, autoradiography following SDS-PAGE revealed a radiolabeled band corresponding to the molecular weight of NfsR only in reactions containing both NfsS and ATP, providing direct evidence that NfsS phosphorylates NfsR. No phosphorylation signal was observed in control reactions lacking NfsR, confirming the specificity of this phosphorylation event (Fig. 2B). As a positive control, we validated the assay conditions by demonstrating PrkC-mediated phosphorylation of its known substrate GpsB from *B. subtilis* (30).

Based on sequence alignments with other PSS proteins, the kinase catalytic residue of NfsS is predicted to be N56, while the serine residue at position 51 (S51) in NfsR is the likely phosphorylation site (Fig. S2). To test this, we generated two variants: a kinase-dead mutant (NfsS[N56A]) and a phospho-ablative mutant of the anti-anti-sigma (NfsR[S51A]). After purifying the proteins (Fig. S3A), we performed *in vitro* phosphorylation assays.

As shown in Fig. 2B, the NfsS[N56A] variant was unable to phosphorylate wild-type NfsR. Furthermore, the NfsR[S51A] variant was not phosphorylated by wild-type NfsS. Together, these results demonstrate that NfsS functions as a kinase that directly phosphorylates NfsR at serine 51 *in vitro*.

### NfsS directly interacts with sigma factors SigC and SigE

Because canonical partner-switching systems regulate transcription by controlling sigma-factor availability through direct binding of the cognate anti-sigma factor, we tested whether NfsS interacts with SigC (All1692) or SigE (Alr4249), two group 2 sigma factors with key roles in heterocyst differentiation (20, 21).

**Fig 3:**
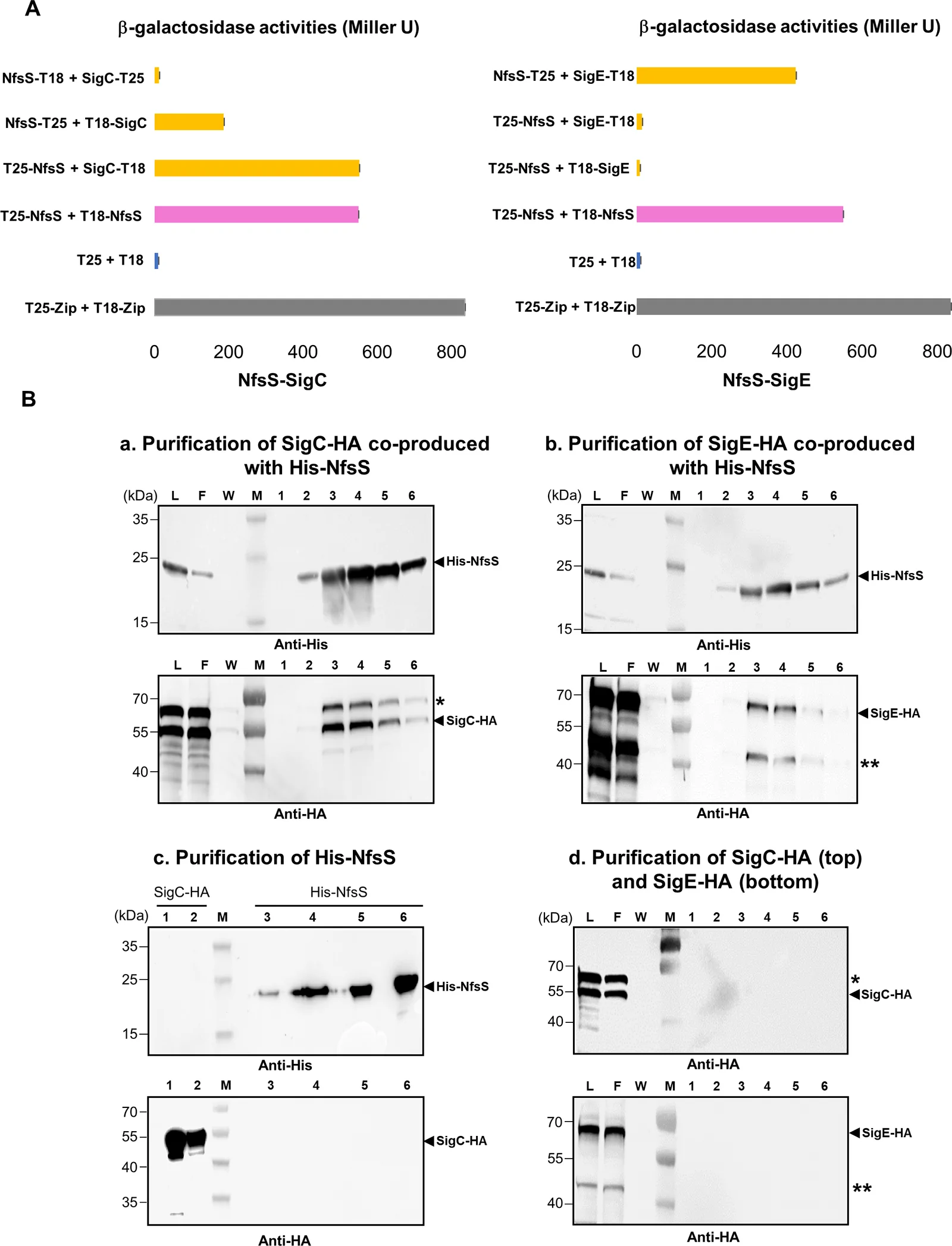
NfsS interacts with SigC and SigE sigma factors. (**A**) Bacterial two-hybrid assay between NfsS and SigC (left) and SigE (right). β-Galactosidase activities were measured as described in Materials and Methods and were expressed in Miller units. Strains producing T18 with T25 served as a negative control. Strains producing T18-Zip and T25-Zip served as a positive control. Data are represented as mean ± SEM, indicated by error bars. (**B**) Pull-down analysis of the interactions between NfsS and SigC or SigE. (**A and B**) Immunoblot analysis of the nickel-affinity co-purification of His₆-tagged NfsS coexpressed with 3× HA-tagged SigC (**A**) or SigE (**B**) in *E. coli*. M, molecular mass marker (kDa); L, loaded cell lysate; F, flow-through; W, wash fraction; 1–6, elution fractions. (**C**) Specificity control for the anti-HA antibody. His-NfsS was produced and purified in the absence of SigC-HA or SigE-HA. Lanes 1 and 2 contain cell extracts corresponding to 0.01 and 0.001 OD units, respectively, of *E. coli* producing SigC-HA and were used as positive controls for anti-HA detection. Lanes 3–6 contain elution fractions corresponding to those in which SigC-HA and SigE-HA were detected in the pull-down assays shown in panels **A** and **B**. M, molecular mass marker (kDa). The membrane was first probed with anti-HA antibodies and then stripped and checked to confirm the complete absence of residual signal before being reprobed with anti-His antibodies. (**D**) Negative controls showing the nickel-affinity purification of HA-tagged SigC and SigE produced in the absence of His-NfsS, using the same procedure as in panels **A** and **B**. M, molecular mass marker (kDa); L, loaded cell lysate; F, flow-through; W, wash fraction; 1–6, elution fractions. The antibodies used for detection are indicated below each blot. The calculated molecular masses of His₆-NfsS, SigC-HA, and SigE-HA are approximately 21, 51.1, and 49 kDa, respectively. Arrows indicate the corresponding immunoreactive bands. His-NfsS, SigC-HA, and SigE-HA migrate slightly above their calculated molecular masses under the SDS-PAGE conditions used. The asterisk indicates a slower-migrating SigC-HA-reactive species detected in the elution fractions. This species was not detected when His₆-NfsS was purified from cells not producing SigC-HA. The double asterisk indicates a degradation product of SigE-HA.

We employed a bacterial adenylate cyclase two-hybrid (BACTH) assay to evaluate potential protein-protein interactions (31). NfsS, SigC, and SigE were fused to the T25 or T18 fragments of *Bordetella pertussis* adenylate cyclase in several possible pairwise combinations and co-transformed into the *E. coli* BTH101 strain, which lacks endogenous adenylate cyclase activity (31). The interaction was evaluated by measuring β-galactosidase activity, with the Zip/Zip pair serving as a positive control and the T25-T18 empty vector pair as a negative control (31). As shown in Fig. 3A, strains co-expressing NfsS with either SigC (NfsS-T25/T18-SigC and T25-NfsS/SigC-T18) or SigE (NfsS-T25/SigE-T18) exhibited β-galactosidase activity well above background and approaching that of the positive control, suggesting strong interactions. Furthermore, this experiment indicated that NfsS can form dimers (or oligomers) (Fig. 3A).

To corroborate the interactions detected in the BACTH assay, we performed affinity pull-down experiments. His_6_-tagged NfsS was coexpressed in *E. coli* with HA-tagged SigC or SigE (Fig. S3B and C), and cell lysates were subjected to nickel-affinity chromatography. SDS-PAGE followed by immunoblotting with anti-His and anti-HA antibodies revealed co-elution of both SigC (Fig. 3B.a, Fig. S4) and SigE (Fig. 3B.b, Fig. S5) with His-NfsS. To assess the specificity of the anti-HA signal, His-NfsS was purified from cells expressing neither HA-tagged sigma factor. NfsS was detected in the eluate with the anti-His antibody, whereas no signal was observed with the anti-HA antibody (Fig. 3B.c, Fig. S6), indicating that the latter did not detect endogenous *E. coli* proteins under the pull-down conditions. Of note, the slower-migrating anti-HA-reactive band detected in the SigC-HA pull-down elution fractions (indicated by an asterisk in Fig. 3B.a) was absent when His-NfsS was purified under the same conditions from cells not producing SigC-HA (Fig. 3B.c; Fig. S6). This observation supports its assignment to a slower-migrating SigC-HA species rather than to an endogenous *E. coli* protein non-specifically retained during purification.

Furthermore, to exclude nonspecific interactions of SigC-HA and SigE-HA with the nickel columns, control pulldowns were performed using SigC-HA and SigE-HA produced alone in the absence of His-NfsS. Neither sigma factor was detected in the eluted fractions, confirming that their retention was dependent on NfsS (Fig. 3B.d; Fig. S7).

Together, these data support the conclusion that NfsS physically associates with SigC and SigE, consistent with its predicted function as an anti-sigma factor within a PSS regulatory module.

### The NfsR anti-anti sigma factor is essential for cell differentiation and diazotrophic growth

**Fig 4:**
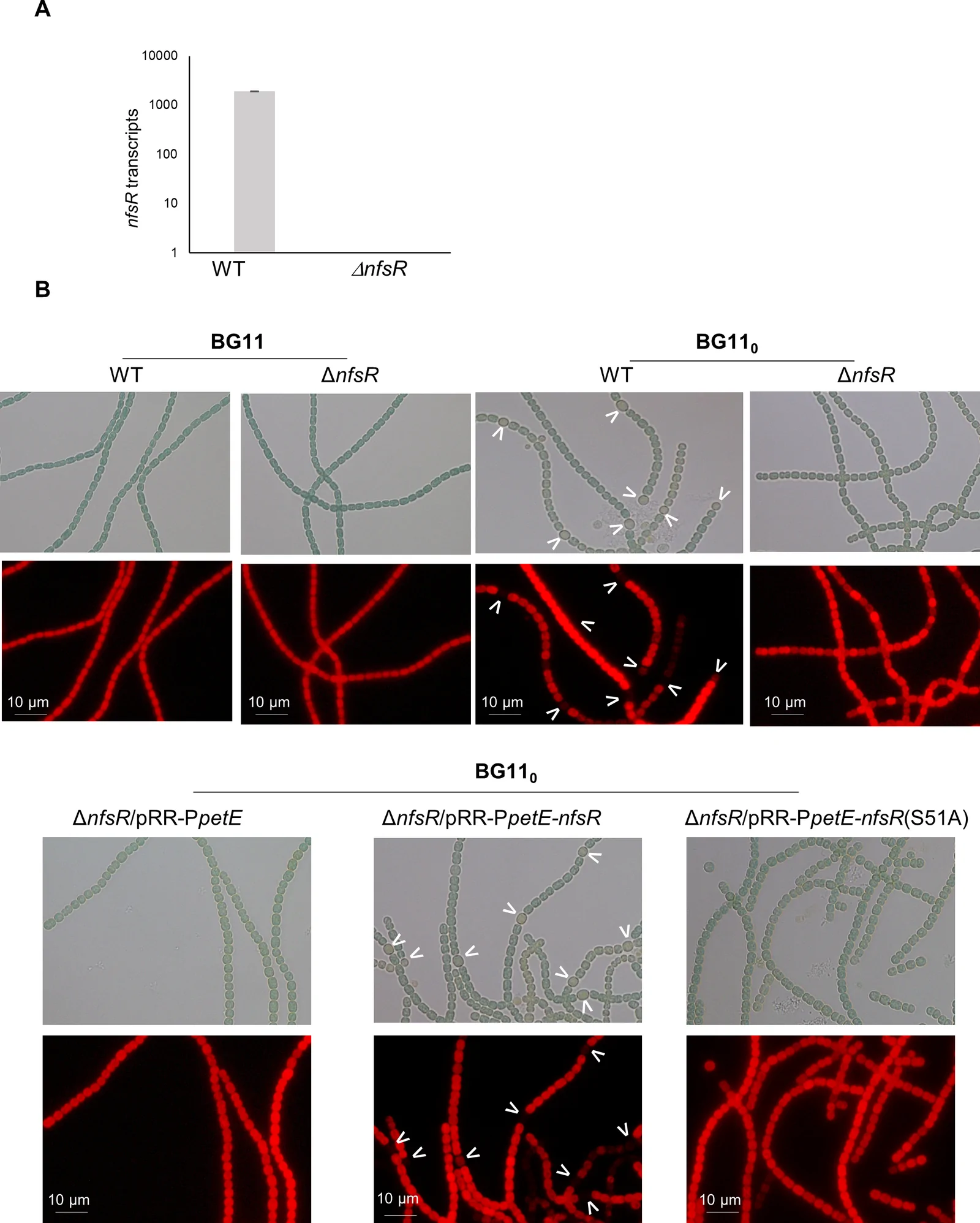
The *nfsR* gene is required for heterocyst differentiation in *Anabaena*. (**A**) RT-qPCR analysis of *nfsR* transcript abundance in the wild-type and Δ*nfsR* strains. The amount of *nfsR* cDNA was quantified in the wild-type (WT) and fully segregated Δ*nfsR* mutant and normalized to the amount of total RNA used for reverse transcription. The results are expressed as the amount of *nfsR* cDNA per microgram of total RNA. Data represent the mean ± SEM of three independent biological replicates. Complete segregation of the Δ*nfsR* mutant was independently confirmed by genomic PCR (Fig. S8A). (**B**) Heterocyst differentiation in the Δ*nfsR* mutant and derivative strains producing wild-type or phosphoablative NfsR. Bright-field images (top) and autofluorescence images (bottom) of the indicated *Anabaena* strains, including the Δ*nfsR* mutant, the mutant carrying a wild-type copy of *nfsR*, and the mutant producing the phosphoablative NfsR variant. Cells grown in BG11_0_ medium were imaged 24 h after combined-nitrogen step-down. White arrows indicate heterocysts.

These results point to a potential regulatory role for the Nfs system in heterocyst formation or function. To test this, we generated a Δ*nfsR* mutant using a CRISPR/Cpf1 approach (32). Complete segregation was confirmed by PCR analysis of the genomic context (Fig. S8A) and by qRT-PCR showing the absence of *nfsR* transcripts (Fig. 4A); 24 h after transfer to nitrogen-depleted medium, the mutant failed to form heterocysts (Fig. 4B, upper panel) and was unable to grow using N_2_ as the sole nitrogen source (Fig. S8A). Thus, NfsR is necessary for the initiation of heterocyst formation and for enabling nitrogen-fixing growth.

Genetic complementation confirmed that NfsR is required for heterocyst differentiation. Introduction of a wild-type copy of *nfsR* into the Δ*nfsR* mutant restored differentiation 24 h after nitrogen step-down, demonstrating that the mutant phenotype results from the loss of functional NfsR (Fig. 4B, lower panel).

To further investigate the NfsR mechanism, we produced a phosphoablative variant of NfsR in the Δ*nfsR* mutant designed to mimic the dephosphorylated state. This approach was based on characterized partner-switching systems in *B. subtilis*, in which alternating phosphorylation and dephosphorylation of the anti-anti sigma factor is required for signal transmission and sigma-factor release from the kinase (i.e., from the anti-sigma factor) (33, 34). We therefore hypothesized that locking NfsR in a dephosphorylation-mimicking state would impair its function.

Consistent with this hypothesis, the phosphoablative NfsR variant failed to restore heterocyst differentiation in the Δ*nfsR* mutant (Fig. 4B, lower panel). This lack of complementation was unlikely to result from markedly reduced protein stability or accumulation, as the same variant produced in *E. coli* accumulated to levels comparable to those of wild-type NfsR and exhibited similar electrophoretic mobility (Fig. S3A). Thus, the complementation behavior of the NfsR variant mirrors that reported for established partner-switching systems and provides further support for the classification of the Nfs system as a partner-switching system.

### The Nfs PSS directs a pleiotropic regulatory network linking metabolism and heterocyst development

Because loss of NfsR abolishes cell differentiation, we investigated its regulon. Given that NfsR acts as the essential positive regulator promoting sigma factor release, this analysis provides the most direct assessment of genes under PSS control.

We assessed the expression of genes associated with heterocyst function by extracting RNA from both the wild-type strain and the *nfsR* mutant under conditions of combined nitrogen deprivation, followed by qRT-PCR analysis at key developmental time points.

**Fig 5:**
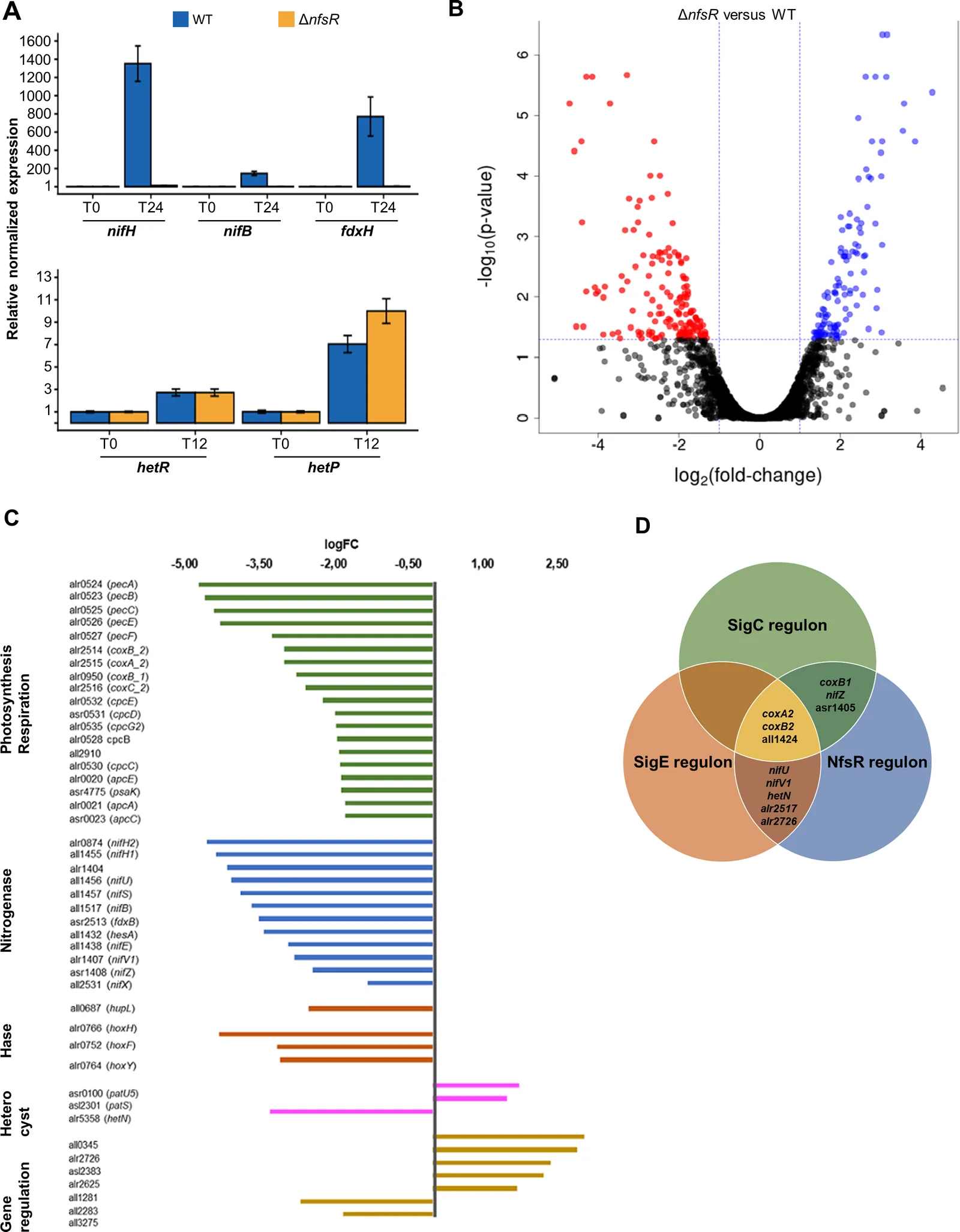
Differential gene expression in the *nfsR* mutant versus wild-type. (**A**) Gene transcription analyzed by qRT-PCR. Each sample was measured in triplicate, and the standard deviation is indicated by error bars. The indicated time refers to the incubation period following combined nitrogen starvation. For each time point, the results are shown as paired bars, with WT and ΔnfsR backgrounds represented by blue and orange bars, respectively. Data are represented as mean ± SEM, indicated by error bars. The values obtained at time 0 were set to 1. (**B**) RNA-seq analysis of the WT and *nfsR* mutant. The volcano plot displays the statistical significance [−log_10_₁(*P*-value)] against the magnitude of change [log_2_(fold change)] for each gene. Significantly upregulated (log_2_FC > 1, adjusted *P*-value < 0.05; blue) and downregulated (log_2_FC < −1, adjusted *P*-value < 0.05; red) genes are highlighted. Gray points represent genes with no significant differential expression. The dashed lines indicate the thresholds for significance (horizontal: adjusted *P*-value = 0.05; vertical: |log_2_FC| = 1). (**C**) Fold change of representative genes from RNA-seq analysis grouped by functional category. The bar graph shows the log_2_(fold change) of selected significantly differentially expressed genes in the *nfsR* mutant compared to the wild-type (WT) strain. Genes were chosen based on statistical significance (adjusted *P*-value < 0.05) and grouped into functional categories to highlight key biological processes affected by the *nfsR* mutation. Error bars represent the standard error of the mean (SEM) from biological replicates. (**D**) Venn diagram illustrating the overlap between genes differentially regulated in an *nfsR* mutant (this study, blue) and the published regulons of the sigma factors SigE (orange) and SigC (green). The significant overlap between the NfsR and SigE/SigC regulons suggests a potential functional interaction or co-regulation within a common stress response network.

As shown in Fig. 5A, *nifH* (encoding a nitrogenase subunit), *nifB* (required for FeMo cofactor synthesis), and *fdxH* (encoding the heterocyst-specific [2Fe-2S]-ferredoxin (35)) were strongly induced in the wild-type strain 24 h after nitrogen step-down, corresponding to the time required for mature heterocyst formation. In contrast, these transcripts were undetectable in the *nfsR* mutant, consistent with its failure to form functional heterocysts.

We next examined the expression of two genes involved in early heterocyst development: *hetR*, which encodes the master regulator HetR (36), and *hetP*, a HetR-regulated gene essential for developmental commitment (37). Transcript levels were measured 12 h after nitrogen deprivation, the peak induction time for *hetP*. Both genes were expressed at similar levels in wild-type and mutant strains (2.7-fold change for *hetR* in both strains and 6.9-fold change in wild-type vs 9.9-fold change in the mutant for *hetP*) (Fig. 5A). As both *hetR* and *hetP* expression were induced in the mutant, we conclude that the mutant phenotype is not due to impaired induction of these known early developmental genes. Therefore, expression of these known early genes is not sufficient for differentiation in the Δ*nfsR* mutant, indicating that NfsR controls a critical checkpoint that operates in parallel to *HetR/HetP* and represents an essential bottleneck for the initiation of heterocyst formation.

To investigate the role of NfsR (and thus the PSS) on a global transcriptional scale, we performed RNA-seq on WT and Δ*nfsR* strains 24 h after nitrogen step-down. The volcano plot in Fig. 5B reveals that 276 genes were differentially expressed in the mutant, with 155 positively regulated by *nfsR* and 121 negatively regulated (Table S2). Figure 5C summarizes the expression patterns of genes belonging to specific functional classes, and complete results are provided in Table S2.

Consistent with the qRT-PCR results, the *nifH*, *nifB*, and *fdxH* genes were among those with significantly reduced expression, validating our global approach (Fig. 5C). This category also included many other genes essential for nitrogenase synthesis or function (highlighted in blue in Fig. 5C), as well as genes encoding uptake hydrogenase, produced specifically in heterocysts and functioning in tandem with nitrogenase (marked in orange in Fig. 5C). Additionally, several genes involved in photosynthesis (e.g., phycobilisome synthesis genes *pec* and *cpc*, and photosystem 1 subunit *psaK*) and respiration (*cox* genes) were differentially expressed (Fig. 5C).

Two genes regulating heterocyst patterning were also affected (marked in pink in Fig. 5C): *patS* (38), which produces a diffusible morphogen inhibiting HetR activity, was overexpressed by an average factor of 2. In contrast, *hetN* (39), responsible for the second inhibitory morphogen, was underexpressed by a factor of 11 (Fig. 5C).

The mutant also exhibited altered expression in six genes encoding transcriptional regulators (marked in brown in Fig. 5C). Other functional classes of underexpressed genes included those involved in primary metabolism, transporters, and a contractile injection system (Table S2). Numerous overexpressed genes were of unknown function (Table S2). Together, these results highlight the broad regulatory influence of NfsR on genes involved in nitrogen metabolism, cellular differentiation, and energy production.

A global transcriptomic study using microarrays previously defined the regulons of the sigma factors SigC and SigE in *Anabaena* (20). Given the interaction between NfsS and these two sigma factors, we compared their transcriptomes, specifically the downregulated genes, with that of NfsR to assess potential overlaps. This comparative analysis revealed a crosstalk between NfsR and both SigC and SigE, particularly involving genes encoding terminal oxidases and nitrogenase (Fig. 5D). The genes shared between the regulons of SigC/SigE and NfsR accounted for 20% of the NfsR regulon.

It should be noted that the sigma factor regulons were analyzed 8 h after the onset of nitrogen deprivation, whereas the NfsR regulon was analyzed at 24 h. This difference in timing may account for the partial overlap observed. Nevertheless, the observation that the most strongly regulated genes in the NfsR regulon (*nif* and *cox*) were also found in the SigC and SigE regulons (Fig. 5C and D) underscores a functional link between the Nfs system and these sigma factors. In summary, our results define NfsR as a critical positive regulator governing the transcriptional program required for heterocyst differentiation.

### A phylum-wide genomic survey reveals enrichment of partner-switching systems in filamentous and heterocyst-forming cyanobacteria

To assess the relevance of PSSs to cyanobacterial cell differentiation, we performed a large-scale *in silico* survey of PSS-encoding genes across 565 complete genomes (see Materials and Methods and Table S1, sheet 4). Genomes contained 2–38 PSS-related genes, with 0–14 anti-anti-sigma (AAS), 0–9 anti-sigma (AS), and 0–22 phosphatase (P) factors. Bifunctional AS-P genes were detected in 69 genomes (12.2%), always as a single copy. Only six genomes lacked at least one PSS functional type (AAS, AS, or P): two *Prochlorococcus marinus*, two *Synechococcus* sp., *Petrachloros mirabilis*, and *Geitlerinema calcuttense* NRMC-F 0142. These included five unicellular species and one filamentous species, suggesting that these organisms may be unable to assemble a complete PSS.

Most PSS homologs (79.4%) contained a single main functional domain (Table S1, sheet 5). We also identified 48 distinct accessory domains (Table S1, sheet 6), of which GAF, DZR, HAMP, and WD40 were the most prevalent, representing 21.4%, 17.1%, 9.8%, and 9.2% of accessory domains, respectively.

**Fig 6:**
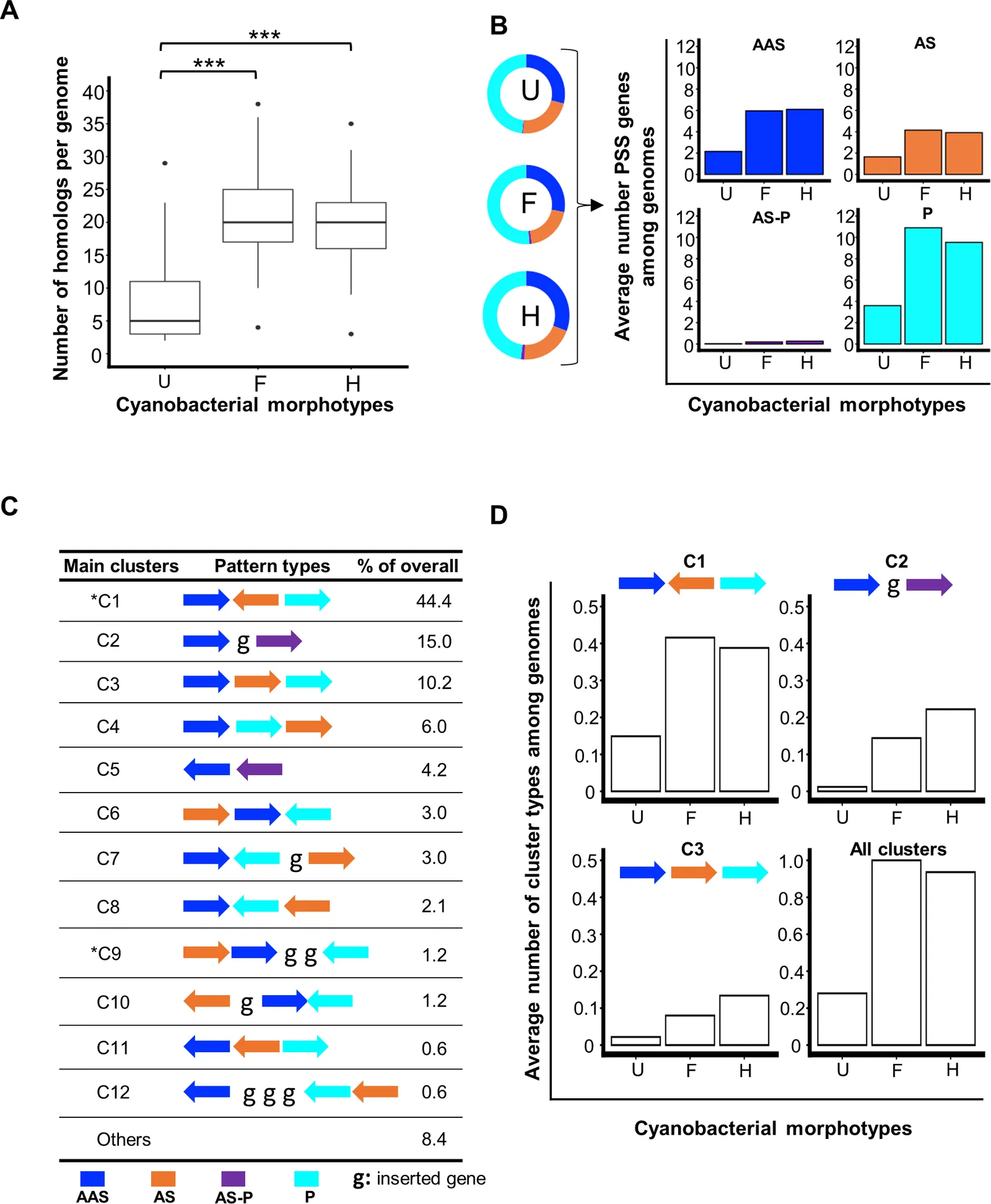
Partner-switching systems are enriched in filamentous and heterocyst-forming cyanobacteria. (**A**) Distribution of the number of PSS gene homologs per genome across cyanobacterial morphotypes. H, heterocyst-forming; F, filamentous non-heterocystous; and U, unicellular organisms. Comparisons were made relative to heterocyst-forming organisms; *** indicates statistical significance by the Wilcoxon-Mann-Whitney test at *P* < 0.001. (**B**) Donut plots showing the relative frequencies of PSS in unicellular, filamentous, and heterocyst-forming organisms (left) and the average number of each PSS type in each morphotype (right). (**C**) Identified functional PSS clusters. Gene organizations (here referred to as pattern types) and their percentages of the total clusters are shown. (**D**) Average numbers of C1, C2, C3, and total functional PSS clusters in each morphotype. Color coding is as follows: AAS, anti-anti-sigma factor, blue; AS, anti-sigma factor, orange; AS-P, bifunctional anti-sigma/phosphatase protein, violet; and P, Ser/Thr phosphatase, cyan. Inserted non-PSS genes are denoted by g. *, cluster types present in *Anabaena* PCC 7120.

We next compared PSS gene distribution across unicellular (U), filamentous (F), and heterocyst-forming (H) cyanobacteria. F and H genomes encoded more PSS homologs than U genomes (Fig. 6A), and this pattern held for all PSS classes (AAS, AS, AS-P, and P; Fig. S4), suggesting that expanded PSS repertoires, potentially through gene duplication, are associated with filamentous and heterocystous lifestyles.

Across the 565 genomes, we identified 7,491 PSS genes, including 29.7% AAS, 20.5% AS, 0.9% AS-P, and 49.3% P. These proportions were similar across morphotypes (U, F, and H) (Fig. 6B, left). However, the mean number of PSS genes per genome was 2.4-fold to 9.4-fold higher in F and H than in U organisms, depending on PSS type (Fig. 6B, right; Table S1, sheet 7). Similar trends were observed across cyanobacterial orders (Table S1, sheet 8). Together, these results indicate that F and H cyanobacteria are enriched in PSS genes, likely reflecting adaptation to diverse environmental signals.

We then searched for functional PSS clusters containing all three PSS components. As defined in Materials and Methods, this analysis identified 333 clusters grouped into 12 categories (C1–C12) based on mirror-cluster patterns (e.g., C1: AAS[+]P[−]AS[+] and its opposite-strand counterpart AS[−]*P*[+]AAS[−]), plus an “Other” category (Table S1, sheets 9 and 10). In total, 35 distinct functional clusters were found, 24 of which contained inserted non-PSS genes, with up to three insertions per cluster (e.g., C7 and C9). The *nfs* PSS cluster of *Anabaena* (C9) was also present in three other heterocystous organisms—*Anabaena* sp. FACHB-595, *Anabaena* sp. FACHB-709, and *Nostoc parmelioides* FACHB-3921—supporting a role in heterocyst differentiation. The most abundant clusters were C1 (44.4%), C2 (15.0%), and C3 (10.2%) (Fig. 6C; Table S1, sheet 10).

Finally, the average number of cluster types per genome, including C1–C3 and total PSS clusters, was approximately 3.5-fold higher in F and H than in U organisms (Fig. 6D; Table S1, sheet 12). Together, these findings support a link between the expansion of functional PSS clusters and the evolution of multicellularity and cell differentiation in cyanobacteria.

## DISCUSSION

**Fig 7:**
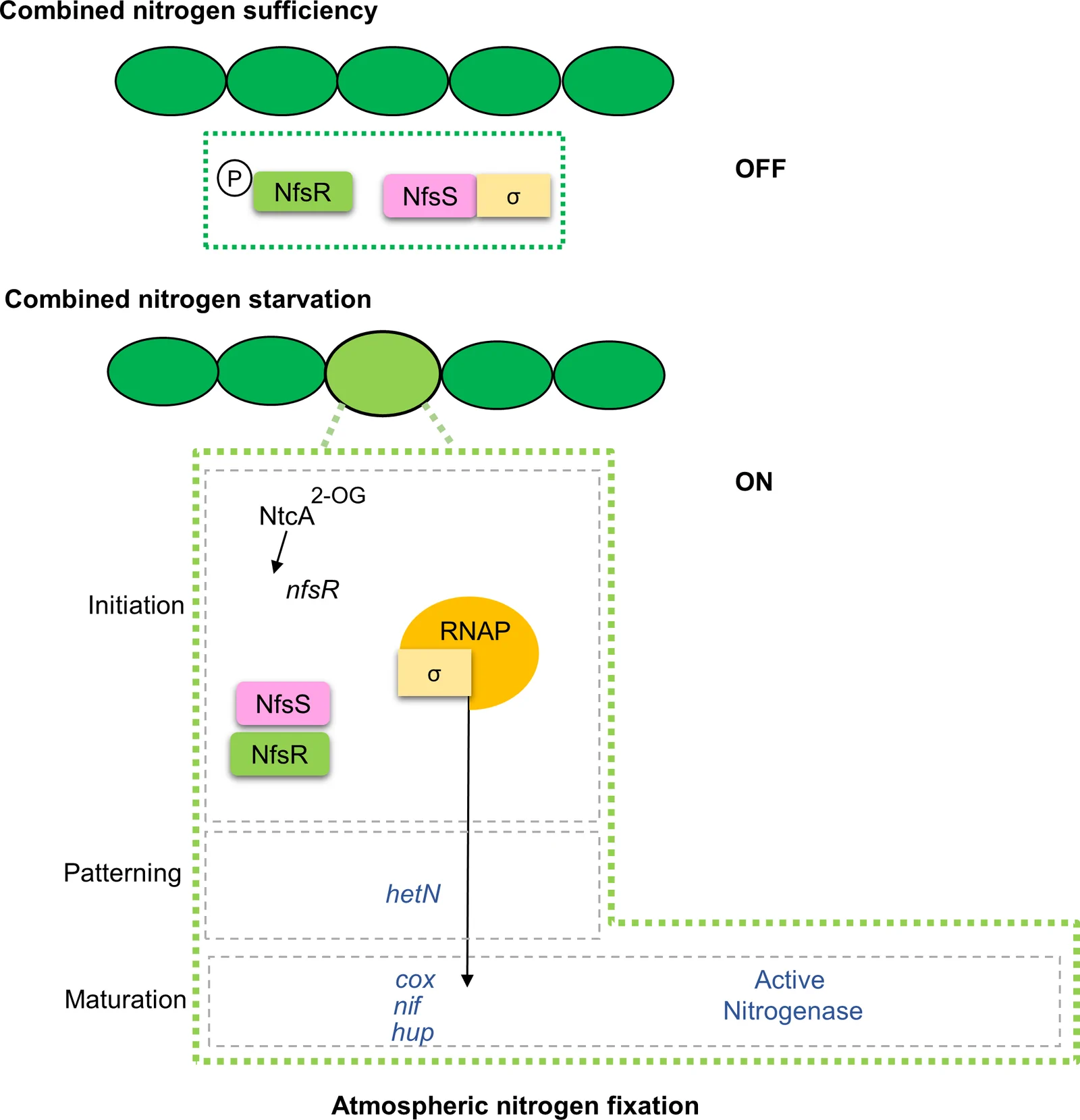
A schematic model for the partner-switching mechanism regulating heterocyst differentiation in *Anabaena*. In the presence of combined nitrogen (upper panel), the anti-sigma factor NfsS sequesters the sigma factors (SigC and SigE), maintaining them in an inactive state. In response to combined nitrogen starvation (lower panel), an unknown signal activates the anti-anti-sigma factor NfsR. Activated NfsR binds and inhibits its cognate anti-sigma factor, NfsS. This partner switch releases the sigma factors, allowing them to direct the transcription of target genes. RNA-seq analysis indicates that genes involved in heterocyst formation (e.g., *patS* and *hetN*) and function (e.g., *nifH* and *hupL*) are among those upregulated upon sigma factor release. A possible role for the Nfs system in vegetative cells during diazotrophic growth, as discussed in the text, is not depicted in this model.

Heterocyst differentiation in *Anabaena* requires extensive transcriptional reprogramming; however, the regulatory mechanisms governing the developmental sigma factors SigC and SigE have remained unclear. Here, we identify a canonical partner-switching system that provides this missing regulatory layer. The anti-sigma factor NfsS interacts with SigC and SigE and phosphorylates its cognate antagonist NfsR, while loss of NfsR abolishes heterocyst formation and diazotrophic growth. Together, these findings define the Nfs module as a molecular checkpoint that couples nitrogen step-down to sigma factor control during developmental commitment (Fig. 7). More broadly, our results expand the known developmental roles of partner-switching systems beyond the canonical sporulation pathway of Firmicutes and identify, to our knowledge, the first such system directly implicated in terminal cell differentiation in a gram-negative bacterium. In doing so, they reveal partner switching as a regulatory principle linking environmental input to cell fate control in a multicellular bacterial lineage.

Our structural and biochemical analyses further indicate that this cyanobacterial module operates through a mechanism closely related to that of the classical SpoIIAA-SpoIIAB partner-switching system. AlphaFold predicts that NfsR and NfsS adopt the characteristic STAS and serine phospho-acceptor folds of these systems, and our *in vitro* assays support a corresponding phospho-transfer mechanism (Fig. 2). Thus, despite the deep phylogenetic distance between cyanobacteria and Firmicutes, a conserved regulatory logic appears to have been redeployed to control a distinct developmental transition.

In *B. subtilis*, the non-phosphorylatable anti-anti-sigma factor SpoIIAA-S58A does not restore productive sporulation, despite behaving as a constitutively active form of SpoIIAA (33). This apparent paradox reflects the fact that dephosphorylated SpoIIAA promotes the induced release of σF from SpoIIAB, but subsequent phosphorylation of SpoIIAA is required to reset the partner-switching cycle, converting SpoIIAA into a form that no longer sequesters SpoIIAB and allowing SpoIIAB to re-enter the inhibitory cycle (34). Thus, SpoIIAB kinase activity on SpoIIAA contributes both to σF activation and to the re-establishment of σF inhibition during sporulation (34).

The failure of NfsR-S51A to restore heterocyst differentiation (Fig. 4B) therefore parallels the SpoIIAA-S58A phenotype and suggests that NfsR phosphorylation may be required for productive progression through the developmental pathway, rather than merely serving to inactivate NfsR. The NfsR-S51A phenotype is therefore consistent with an induced-release model for regulation by the Nfs partner-switching system.

Genetic and transcriptomic evidence place the Nfs system upstream of the partially redundant sigma factors SigC and SigE. Although single *sigC* or *sigE* mutants remain capable of differentiation (17, 20), inactivation of *nfsR* completely blocks heterocyst formation and diazotrophic growth (Fig. 4B; Fig. S8A). Consistent with this phenotype, the transcriptome of the Δ*nfsR* mutant 24 h after nitrogen deprivation shows broad downregulation of late heterocyst genes, including nitrogenase structural genes and other functions required for mature heterocyst activity (Fig. 5). Although the overlap between the NfsR-dependent transcriptome and previously defined SigC/SigE regulons is limited, that difference is likely explained, at least in part, by the distinct developmental stages examined in the different data sets. Even so, the global failure to induce sigma factor-dependent developmental genes strongly supports the view that the Nfs system acts upstream in this regulatory cascade.

The simplest mechanistic model is that NfsR regulates access of SigC and SigE to RNA polymerase by controlling their sequestration by NfsS. In this view, failure to activate NfsR would leave SigC and SigE unavailable for assembly into active holoenzymes, thereby impairing transcription of genes required for differentiation and maturation. This model is parsimonious and is supported by our interaction and phosphorylation data. Importantly, the Δ*nfsR* phenotype is unlikely to result simply from a global loss of HetR activity because transcription of several genes directly activated by HetR, including *hetP*, *hetZ,* and *patA,* was not abolished in the mutant (Fig. 5A; Table S2). At the same time, our results do not exclude the possibility that the Nfs system also acts indirectly by enabling the expression of additional transcriptional regulators needed for progression through the developmental program because several genes encoding putative regulatory factors are downregulated in the Δ*nfsR* mutant. These possibilities are not mutually exclusive, but both support the conclusion that the Nfs system contributes to the transcriptional competence required to enter and sustain heterocyst differentiation.

The extent of the Δ*nfsR* transcriptional defect further suggests that the Nfs system contributes to a broader cellular response to combined nitrogen starvation (Fig. 5). In particular, altered expression of genes encoding phycobilisome components raises the possibility that this system participates in remodeling of the photosynthetic apparatus, potentially in vegetative cells as well as in differentiating cells. This interpretation is consistent with earlier observations that transcripts for phycobilisome-associated genes are largely vegetative-cell specific in *Anabaena variabilis* ATCC 29413 (40) and that, when measured at the filament scale, transcriptional changes occurring within heterocysts have limited impact on total filament transcript abundance (40, 41). At the same time, several studies have reported maintenance of certain photosystem II proteins in heterocysts (42), and the cell type-specific transcriptional regulation of genes encoding photosystem and phycobilisome components remains unresolved. Our findings, therefore, raise the possibility that the Nfs system coordinates not only developmental sigma-factor activity but also broader transcriptional adjustments associated with nitrogen starvation.

Our comparative genomic analysis places this system in a broader evolutionary framework. The major expansion in partner-switching content occurred with the transition from unicellular to filamentous cyanobacteria: although the relative proportions of AAS, AS, AS-P, and P components remained similar across morphotypes, filamentous and heterocyst-forming genomes contained approximately 3-fold to 4-fold more components of each class and 3.3-fold to 3.5-fold more functional clusters than unicellular genomes. This pattern is most consistent with global amplification of an ancestral partner-switching repertoire during the emergence of multicellularity, likely through duplication at both the gene and cluster levels, followed by specialization of distinct modules for developmental functions. In that context, our identification of the *nfs* system assigns heterocyst differentiation as a new developmental output of partner switching. Together with the previously described noncanonical partner-switching module controlling hormogonium development in *N. punctiforme* (9), these data suggest that cyanobacteria have repeatedly recruited partner-switching circuits to govern differentiated cell states. The marked enrichment of partner-switching genes and clusters in filamentous and heterocystous taxa further suggests that these systems diversified to process the broader range of environmental and developmental signals associated with multicellular life, raising the possibility that additional pathways may contribute to other cyanobacterial developmental programs, including akinete formation, multiplane fission, or true branching.

The major next step is to identify the cognate phosphatase that activates NfsR. The all2280 gene, located near *nfsS* and *nfsR* (Table S1), represents a strong candidate for this function and will therefore be a priority for future investigation. Once the phosphatase has been identified, a further objective will be to determine how nitrogen step-down is sensed upstream of the Nfs phospho-switch. Partner-switching phosphatases are activated through diverse signal-sensing mechanisms (3), and defining this signaling route will be essential for understanding how environmental information is converted into a developmental decision. By placing a partner-switching system at the nexus of nitrogen sensing and sigma-factor activation, our work establishes a mechanistic foundation for dissecting how multicellular cyanobacteria couple environmental cues to cell differentiation, with implications for both heterocyst biology and the evolution of developmental regulation in bacteria.

## MATERIALS AND METHODS

### Bioinformatics analysis

#### Data sets

Reference PSS seed proteins (13 in total; Table S1, sheet 1) were identified from the literature and retrieved from UniProt, with priority given to proteins supported by experimental evidence. Genome data for 565 cyanobacterial organisms available as of March 2026 were downloaded from the NCBI FTP site (listed in Table S1, sheet 2). This data set included all genomes at the highest assembly levels, mostly classified by NCBI as “Complete” or “Chromosome” (~71.6% of the total), and when such assemblies were unavailable, genomes were classified as “Scaffold” or “Contig” but restricted to “Reference” or “Representative” status. The downloaded files also included gene annotation features and organismal taxonomic lineages.

Morphological and physiological traits of the cyanobacterial organisms were assigned based on published studies (43–46). The selected organisms spanned the broad diversity of cyanobacteria, covering 22 major taxonomic orders, of which 55.6%, 22.1%, and 22.3% corresponded to unicellular (U), filamentous non-heterocystous (F), and filamentous heterocyst-forming (H) organisms, respectively. The four unclassified cyanobacterial organisms in the data set (three cyanobacterial endosymbionts and *Adonisia turfae* CCMR0081) were arbitrarily assigned to the unicellular category. Hidden Markov model (HMM) profiles for protein domain families (Pfam 38.1; January 2026) were downloaded from the Pfam FTP site.

#### Identification and distribution analysis of PSS homologs and clusters

The HMMER package (47), together with Pfam HMM profiles, was first used to identify seed functional domains in the reference proteins and define their domain organization. Alignments scoring above the Pfam trusted cutoffs (39) yielded 12 reference seed domains (Table S1, sheet 3), six of which were considered main functional domains. The HMMER package and custom Perl scripts were then used to search cyanobacterial genomes for PSS orthologs, requiring each candidate protein to contain at least one main seed domain. In cases of overlapping domain matches, the longest match with the best E value was retained.

Candidate PSS orthologs were further examined to verify their domain architecture, and only proteins containing at least one main domain were retained, except for AAS orthologs, for which only proteins with a strict STAS domain were kept (Table S1, sheets 4 and 5). In-house Perl scripts were subsequently used to identify functional PSS clusters (Table S1, sheet 6). A functional PSS cluster was defined as a genomic region containing homologs representing the three distinct PSS functions (AAS, AS, and P), flanked by PSS genes and containing either no intervening non-PSS gene or at most three inserted non-PSS genes. AS-P homologs were considered to contribute both AS and P functions.

This procedure yielded PSS cluster patterns based on relative gene positions from −5 to + 5 using the AAS gene as the reference point (position 0). Clusters and their mirror configurations on the opposite DNA strand were grouped into 12 classes (C1–C12) (Table S1, sheets 6, 7, and 8). The distribution of PSS homologs and functional PSS clusters across cyanobacterial genomes was analyzed primarily according to morphotype (U, F, and H) (Table S1, sheets 9 and 10). Multiple-sequence alignments were performed with Clustal Omega.

### Strains, plasmids, and primers

The strains, plasmids, and primers used in this study are listed in Tables S3 to S5. The construction of plasmids is described in the supplemental material.

### Growth conditions, conjugation, and heterocyst induction

*Anabaena* and derivatives were grown in BG11 medium at 30°C under continuous illumination (40 µE m^−2^ s^−1^). Concentrations of antibiotics, the induction of heterocysts in media lacking a source of combined nitrogen, and conditions for microscopy were described previously (48).

### Bacterial two-hybrid assays

Bacterial two-hybrid assays followed the method of Karimova et al. (31). The BTH101 strain, co-transformed with T18- and T25-fusion plasmids, was plated on LB containing ampicillin and kanamycin and incubated at 30°C for 2 days. For each assay, 10 colonies were grown in 3 mL of LB with antibiotics and 0.5 mM IPTG overnight at 30°C. β-Galactosidase activity, measured according to a previously described method (49), is reported as means from three independent assays.

### Mutant construction

The *nfsR* mutant was created using the CRISPR-Cpf1 system present on the pCpf1-ccdB (32) or the pMB39 (50) plasmids. We amplified 1 kbp upstream and downstream regions from *Anabaena* gDNA using RP-up-Fwd/Rev and RP-down-Fwd/Rev primers, respectively. These fragments were then cloned between BamHI sites of the pCpf1-ccdB plasmid. We designed spacer sequences as described in (51). Gene-specific Cr-Fwd and Rev primers were annealed at 95°C to generate spacers specific to each gene, which were then ligated into the AarI sites of the respective plasmids. We introduced the editing plasmids into *Anabaena* via conjugation.

Exconjugants were isolated on BG11 plates supplemented with 2.5 µg/mL spectinomycin and streptomycin for eight successive generations, then grown in liquid culture for gDNA extraction. We confirmed the absence of the edited gene and complete segregation of the mutants by PCR on gDNA. Fully segregated clones were plated on BG11 plates without antibiotics and then on plates with 10% sucrose to counter-select the editing plasmid. We verified the loss of the editing plasmid by colony PCR, plating the resulting strain on BG11 supplemented with 2.5 µg/mL spectinomycin and streptomycin. All experiments were conducted on two independent clones.

### Microscopy

Microscopic observations were conducted using a Nikon Eclipse E800 optical microscope with a ×60 magnifying objective. Images were captured with a Nikon DXM 1200 digital camera, equipped with a PE-300lite (CoolLED) light source and controlled via ATC-1 software. Bright-field observations used white light, GFP fluorescence used a FITC filter (Ex 465/495 nm, Dm 505 nm), and photosynthetic pigment fluorescence used a TRITC filter (Ex 540/25, Dm 565).

### RNA preparation, reverse transcription, and quantitative real-time PCR

RNAs were prepared using the Qiagen RNA extraction kit per the manufacturer’s instructions, with an additional TURBO DNase (Invitrogen) digestion to remove DNA contamination. RNA quality was assessed with an Agilent TapeStation and quantified at 260 nm using a NanoDrop 1000 (Thermo Fisher). For cDNA synthesis, 1 µg of total RNA and 0.5 μg of random primers (Promega) were used with GoScript Reverse Transcriptase (Promega) as per instructions. Quantitative real-time PCR (qPCR) was conducted as previously described (48).

### Protein production and purification

The genes encoding His-NfsS, His-NfsR, His-NfsS[N56A], and His-NfsR[S51A] were expressed in *E. coli* BL21(DE3) cells. A 1 L culture was grown at 37°C in LB medium to an OD₆₀₀ of 1.0. Protein production was induced with 0.4 mM IPTG at 16°C overnight.

Cells were harvested by centrifugation and resuspended in 25 mL of lysis buffer (50 mM Tris-HCl, pH 7.4 or pH 8 for NfsR and NfsS, respectively), 300 mM NaCl, 20 mM imidazole, 20% glycerol, and 2 mM β-mercaptoethanol) supplemented with 0.5 mg mL⁻¹ lysozyme and 1 mg DNase I. The suspension was lysed using a French press at 10,000 p.s.i. The lysate was clarified by ultracentrifugation and loaded onto a 1 mL HiTrap Ni-NTA column (Cytiva) using an ÄKTA Prime system. The column was washed with lysis buffer until the A₂₈₀ baseline was stable. Bound protein was eluted with a linear imidazole gradient from 20 mM to 1 M. Eluted fractions were analyzed by SDS-PAGE, and those containing the protein of interest were pooled and desalted into storage buffer (50 mM Tris-HCl [pH 7.4 or pH 8], 100 mM NaCl, 20% glycerol, and 2 mM β-mercaptoethanol) using PD-10 desalting columns (Cytiva). Protein concentration was determined using the Bio-Rad Protein Assay (Bradford method).

### Phosphorylation assays

Proteins (at 0.4 mg/mL for NfsS, 0.5 mg/mL for NfsR, 2 mg/mL for PrkCc, and GpsB) were incubated for 3 h at 25°C in a 15 μL reaction mixture containing 50 mM Tris/HCl, pH 7.5, 1 mM DTT, 50 mM NaCl, 0.1 mM EDTA, 5 mM MgCl_2_, and 0.5 mM [γ-^33^P]ATP (1 μCi). The phosphorylation reaction was stopped by adding 5× SDS-sample buffer to the reaction mixtures before SDS-PAGE analysis. Gels were then dried and exposed overnight to autoradiography.

### Pull-down assay

For pull-down assays, *E. coli* BL21(DE3) Lemo21 cells were transformed with pET-duet_His-NfsS, pCDF_sigC-3HA, or pCDF_sigE-3HA or co-transformed with two plasmids: pET-duet_His-NfsS and either pCDF_sigC-3HA or pCDF_sigE-3HA. A 50 mL culture was grown at 37°C to an OD_600_ of 0.6, and expression was induced with 0.25 mM IPTG at 16°C overnight.

Cells were harvested and lysed in 10 mL of pull-down buffer (50 mM Tris-HCl, pH 8.0, 400 mM NaCl, 20 mM imidazole) supplemented with 0.5 mg/mL lysozyme and 20 µg/mL DNase, using a French press. The clarified lysate was incubated with 1 mL of Ni-NTA resin. The resin was washed extensively with pull-down buffer to remove unbound proteins. Specifically, bound proteins were eluted with pull-down buffer containing 500 mM imidazole. Elution fractions (1–6) were resolved on a 4%–20% gradient polyacrylamide gel (NuSep) and analyzed by western blot using α-His (Qiagen) and α-HA antibodies (Enzo Life Science).

### RNA sequencing analysis

*Anabaena* cultures (100 mL in BG11 medium) were grown to an OD_750_ of 0.6–0.8. Cells were harvested and washed three times with nitrogen-free BG11_0_ medium. The washed cultures were then divided into three biological replicates of 30 mL each. After 24 h of nitrogen starvation, total RNA was extracted from each 30 mL replicate. RNA integrity (RIN ≥ 8) was verified on an Agilent 2100 Bioanalyzer. rRNA was depleted with the Ribo-Zero Bacteria kit (Illumina), and strand-specific libraries were prepared at the TGML platform (Transcriptomics and Genomics Marseille-Luminy, Aix-Marseille Université) according to the TruSeq Stranded mRNA protocol. Briefly, 1 µg RNA was fragmented (94°C, 8 min), reverse-transcribed with SuperScript IV, and second-strand cDNA was synthesized incorporating dUTP to preserve strand information. After end-repair, A-tailing, and adapter ligation, 13 cycles of PCR enrichment were applied. Libraries were quantified by Qubit and qPCR, pooled equimolarly, and sequenced on an Illumina NovaSeq 6000 (2 × 150 bp, ~30 million PE reads per sample).

Illumina FASTQ reads were aligned to the *Anabaena* (*Nostoc* PCC7120) genome (NCBI, no. GCF_000009705.1; release date, 7 January 2024) with STAR v2.7.1a; multimapping reads were excluded (-m 1). Uniquely mapped fragments (paired reads) were counted at the gene level with featureCounts v1.6.4, considering only fragments mapped to the proper strand (-s 2). Raw counts were imported into edgeR (R v3.6) for library-size normalization; genes with <1 FPKM across all replicates were removed. Differential expression was calculated using the exact-test function in edgeR, applying a Benjamini-Hochberg FDR threshold of <0.01. Significant transcripts were classified as up- or down-regulated in the mutant versus the wild type, yielding two expression groups. Only genes having a significant log fold change >2.5 with a Benjamini-Hochberg-corrected *P*-value <0.01 were considered in the study.

### Statistical analysis

For β-galactosidase activities, the values presented are means of three independent assays, each containing 10 independent colonies. The results are expressed as the means ± standard deviation. For qRT-PCR, the 16S RNA gene was used as a reference for normalization. All measurements were carried out in triplicate, and a biological triplicate was performed for each point. The amplification efficiencies of each primer pair were 80%–100%. Biological replicates were log_2_ transformed and compared by a two-tailed unpaired *t*-test. Data are reported as mean ± SEM of normalized values, rescaled so that the control group means equal 1.

For genomic analyses, Wilcoxon-Mann-Whitney tests, one-sample *t*-tests, and boxplots and distribution graphs were performed in R (52). Normality was assessed using the Shapiro-Wilk test with a 5% significance threshold. When the data followed a normal distribution, Student’s *t*-test was applied. Otherwise, the non-parametric Wilcoxon-Mann-Whitney test was used, with a 5% significance threshold.

## Data Availability

The data discussed in this publication have been deposited in NCBI’s Gene Expression Omnibus (53) and are accessible through GEO Series accession number GSE321668 (https://www.ncbi.nlm.nih.gov/geo/query/acc.cgi?acc=GSE321668).

## SUPPLEMENTAL MATERIAL

Supplemental Tables and Figures mbio.01685-26-s0001.docx Tables S3 to S5; Fig. S1 to S8.

Table S1 mbio.01685-26-s0002.xlsx Genomic analysis.

Table S2 mbio.01685-26-s0003.xlsx RNA seq data.

ASM does not own the copyrights to Supplemental Material that may be linked to, or accessed through, an article. The authors have granted ASM a non-exclusive, world-wide license to publish the Supplemental Material files. Please contact the corresponding author directly for reuse.
