# Supplementary material for: A conserved partner-switching system controls terminal differentiation in multicellular cyanobacteria
Source: mBio. 2026 Sep 3;17(10):e01685-26. doi: 10.1128/mbio.01685-26 (PMC13712970; doi:10.1128/mbio.01685-26)
Supplement: Supplementary file 1 [file mbio.01685-26-s0001.docx]

**Supplementary Material**

**Plasmid construction:**

All the primers used for plasmid constructs are listed in Table S3. All the cloning steps were carried out in DH5α *E. coli* strain, and In-fusion (Takara) steps were carried out using Cloning Enhancer® based method (Takara) according to the manufacturer's instructions. All the PCR amplification steps were performed with CloneAmp® (Takara) polymerase mix. All the recombinant plasmids were analyzed by sequencing. Plasmid sequences and maps are **available from the corresponding author upon request.**

Gene fusion

*PnfsR*::*gfp*: the *nfsR* promoter region (−1000 to +3 from ATG) was amplified from *Anabaena* gDNA using 2283 Prom-gfp_Fw/Rv primers, then inserted into pRR-duet between SphI and BseRI by In-Fusion.

Complementation assays

pRR/*petE*-*nsfR*: the open reading frame of *nsfR* was amplified from Anabaena gDNA using the 2283oe_Fw/Rv in-fusion primers and cloned between the NdeI restriction sites in the pRR002 plasmid.

pRR/*petE*-*nsfR*(S51A) : Mutations in *nfsR* were introduced by site-directed mutagenesis on the pRR/*petE*-*nsfR* plasmid. Serine 51^st^ coding sequence was changed to Alanine codon using all2283 mut fw and all2283 mut rv primers.

Protein production and pull-down

pET28/*nfsR*: the coding sequences of *nfsR* and were amplified from Anabaena gDNA using the all2283-NdeI_fw/ all2283-EcoRI_rv primers and then cloned between the NdeI and EcoRI restriction sites in the pET28a vector.

pET28/*nfsR*[S51A]: Mutations in *nfsR* were introduced by site-directed mutagenesis on the pET28/*nfsR* plasmid. Serine 51^st^ coding sequence was changed to Alanine codon using all2283 mut fw and all2283 mut rv primers.

pET-duet/*nfsS*: the open reading frame of *nfsS* was amplified from Anabaena gDNA using all2284-BamHI-fw/all2284-PstI-rv primers, then cloned in pET-duet vector between BamHI and PstI sites.

pET-duet/*nfsS*[N56A]: Mutations in *nfsS* were introduced by site-directed mutagenesis on the pET-duet/*nfsS* plasmid. Asparagine 56^th^ coding sequence was changed to Alanine codon using all2284 mut fw and all2284 mut rv primers.

pCDF/*sigC*-HA: the open reading frame of *sigC* was amplified from Anabaena gDNA using (pCDF)sigC_Fw2/(3HA) sigC_Rv Rv primers, then cloned by In-Fusion in pCDFduet vector between NdeI and XhoI, in the reading frame with the C-terminal HA-tag, amplified from the pCDF *hetL*-HA plasmid (1) using the (sigC)3HA_Fw and (pCDF)3HA_Rv primers.

pCDF/*sigE*-HA: the open reading frame of *sigE* was amplified from Anabaena gDNA using (pCDF)sigE_Fw2/(3HA)sigE_Rv primers, then cloned by In-Fusion in pCDFduet vector between NdeI and XhoI, in the reading frame with the C-terminal HA-tag, amplified from the pCDF hetL-HA plasmid using the (sigE)3HA_Fw and (pCDF)3HA_Rv primers.

Bacterial two-hybrid

The *nfsS*, *sigC,* and *sigE* genes were amplified from genomic DNA of *Anabaena* using the primers listed in Table S3. The recombinant plasmids were constructed by inserting gene sequences of interest into pKT25 (T25 at N-terminus), pKNT25 (T25 at the C-terminus), pUT18 (T18 at the C-terminus), and PUT18C (T18 at the N-terminus) (Karimova et al. 1998), between Pst1 and EcoR1 restriction sites of the BACTH plasmids

Sequence of the 3xHA tag:

Nucleotide sequence:

TACCCATACGATGTTCCAGATTACGCTGGATATCCCTATGACGTGCCCGACTATGCCGGTTACCCGTACG ATGTCCCGGACTACGCCGGCGGCGGCGGCTCA

Amino acid sequence:

YPYDVPDYAG YPYDVPDYAG YPYDVPDYA GGGGS

**Table S3**

***Anabaena* and *E. coli* strains**

| **Strains** | **Description/genotype** | **Source/reference** | |  |
| --- | --- | --- | --- | --- |
| ***Anabaena* strains** |  |  | |  |
| *Anabaena* PCC 7120 | Wild-type strain (WT) | Pasteur Institute Collection | |  |
| Δ*nfsR* | Δall2283; Derives from *Anabaena* | This study | |  |
| WT/ P*_nfsR_*::*gfp* | WT/ P*_nfsR_*::*gfp* Wild type strain containing the pRR-2MCS-Pall2283::*gfp* plasmid | This study | |  |
| Δ*nfsR* / pRR-P*petE* | Δall2283 strain containing the pRR-P*petE* plasmid | This study | |  |
| Δ*nfsR* / pRR-P*petE-nfsR* | Δall2283 strain containing the pRR-P*petE-*all2283 plasmid | This study | |  |
| Δ*nfsR* / pRR-P*petE-nfsR* (S51A)  ***E. coli* strains** | Δall2283 strain containing the pRR-P*petE-*all2283(S51A) plasmid | This study | |  |
|  |  |  | |  |
| DH5α | *fhuA2 lac(del)U169 phoA glnV44 Φ80' lacZ(del)M15 gyrA96 recA1 relA1 endA1 thi-1 hsdR17* | NEB | |  |
| BL21 DE3 | *fhuA2 [lon] ompT gal (λ DE3) [dcm] ∆hsdS λDE3 = λsBamHIo ∆EcoRI-int::(lacI::PlacUV5::T7 gene1) i21∆nin5* | NEB | |  |
| BTH101 | F^−^, cya-99, araD139, galE15, galK16, rpsL1, hsdR2, mcrA1, mcrB1 | (2) | |  |
| AM5501 | DH5α strain containing both pRL443 and pAM5505 plasmids for conjugation | (3) | |  |
| BL21DE3/*pnfsS*_  BL21DE3/*pnfsR :*  BL21DE3/*pnfsS*[N56A]  BL21DE3/*pnfsR*[S51A]  BL21DE3/*psigC*  BL21DE3/*psigE* | BL21DE3 strain containing the pET-duet/*nfsS* plasmid  BL21DE3 strain containing the pET28/*nfsR* plasmid  BL21DE3 strain containing the pET-duet/*nfsS*[N56A]plasmid  BL21DE3 strain containing the pET28/*nfsR*[S51A] plasmid  BL21DE3 strain containing the pCDF-duet_*sigC*-3HA plasmid  BL21DE3 strain containing the pCDF-duet_*sigE*-3HA plasmid | This study  This study  This study  This study  This study  This study | |  |
| BTH101/pKT25-*nfsS* | BTH101 containing the plasmid pKT25-*nfsS* | This study | | This study |
| BTH101/pUT18-*nfsS* | BTH101 containing the plasmid  *nfsS*-pUT18 | This study | | This study |
| BTH101/pKNT25-*nfsS* | BTH101 containing the plasmid  *nfsS*-pKNT25 | This study | | This study |
| BTH101/pUT18-*sigE* | BTH101 containing the plasmid *sigE*-pUT18 | This study | | This study |
| BTH101/pUT18-*sigC* | BTH101 containing the plasmid *sigC*-pUT18 | This study |  |  |
| BTH101/pKNT25-sigC | BTH101 containing the plasmid *sigC*-pKNT25 | This study |  |  |
| BTH101/pUT18C-*sigC* | BTH101 containing the plasmid pUT18C-*sigC* | This study |  |  |

**Table S4: Plasmids used in this study**

**Plasmids	Description	Source/reference**

pRL443	Ap^R^. RT4 derivative plasmid for conjugation	Addgene	(#[70261](https://www.addgene.org/70261/))	(3)

PAM5505	Cm^R^. pRL623 derivative helper plasmid for conjugation	Addgene	(#[132664](https://www.addgene.org/132664/)) (3)

pAM5411	Sp/Sm^R^. Replicative in *Anabaena*, contains the e*yfp* coding	Addgene	sequence under the P*tac* promoter control	(#[132663](https://www.addgene.org/132663/)) (3)

pRR002	pAM5411 derivative, P*tac* and e*yfp* removed,	(4)	with the copper inducible P*petE* promoter added.	For gene overexpression in the *Anabaena* strain.

pRR-*petE*-*nfsR*	pRR002 containing the all2283 coding sequence	(4)

pRR-*petE*-*nfsR*	pRR002 containing the all2283 coding sequence	(4)

pRR-duet	pAM5411 derivative, P*tac* and e*yfp* removed,	(4)	with two synthetic MCS and promoterless *GFP* gene added.	For transcriptional and translational *GFP* fusions

pET28a	Kn^R^. For his-tagged protein production in *E.coli*	Novagen®

pCDF-duet™	Sp/Sm^R^. For C-terminally HA-tagged protein production	, Merck	in *E. coli*	(#71340)

pET-duet	Ap^R^. For His-tagged protein production	in *E. coli*	Sigma

pCpf1-ccdB	Neo^R^. Replicative in *Anabaena*, Cpf1-based CRISPR	(5)	plasmid with CcdB and SacB counter-selection markers.

pCpf1-*nfsR*	pCpf1-KR-ccdB derivative for all2283 deletion	This study

pET-duet_*nfsS*	pET-duet containing the all2284 gene for expression	This study	in the *E. coli* strain

pET-duet_*nfsS*[N56A]	pET-duet containing the all2284 mutated gene for	This study

expression in the *E. coli* strain

pCDF-*sigC*-3HA	pCDFduet containing the *sigC-*3HA gene fusion	This study

pCDF-*sigC*-3HA	pCDF duet containing the *sigE*-3HA gene fusion	This study

pET28-*nfsR*	pET28a containing the all2283 gene fusion for expression	This study

in *E. coli*

pET28-*nfsR*[N51A]	pET28a containing the all2284 gene fusion for expression	This study

in *E. coli*

pKT25-*nfsS*	pKT25 plasmid containing *nfsS*	This study

pUT18-*nfsS*	pUT18 plasmid containing *nfsS*	This study

pKNT25-*nfsS*	pKNT25 plasmid containing *nfsS*	This study

pUT18-*sigE*	pUT18 plasmid containing *sigE*	This study

pUT18-*sigC*	pUT18 plasmid containing *sigC*	This study

pKNT25- *sigC*	pKNT25 plasmid containing *sigC*	This study

pUT18C-*sigC*	pUT18 plasmid containing *sigC*	This study

**Table S5: Sequence of the primers used in this study**

**PRIMERS**	**5’-3’ Sequence**

**CRISPR/ plasmid construction**

**Protospacers**

Cr1.all2283 fw	AGATATGGTAAAAAAGGCAGACAAAT

Cr1.all2283 rv	ATCTACAACAGTAGAAATTATTTGTCTGCCTTTTTTACCAT

Cr2.all2283 fw 	AATTTCTACTGTTGTAGATGCACTCTGTTCAATCAACGAAC

Cr2.all2283 rv	AGACGTTCGTTGATTGAACAGAGTGC

**Repair template**

RP-all2283 dw_fw	CGGGAGACATATCCGCTTTCAGTCAAGAG

RP-all2283 dow- rv	CGTTGTTGCCATTGCGGATCCTCTCTCCTCAGCAGTTTTTGCCA

RP-all2283 up-fw	ATATCTAGATCTCATGGATCCCTGGTGCTTTCTTTAAAAATGACA

RP-all2283up_rv	GAAAGCGGATATGTCTCCCGATAAATAGAATTTGT

**Deletion strain verification**

Del all2283_fw	ACACCAAGATTATTCAACCTAGTGG

Del all2283_rv	ACTGTTTGCATAAAAGCTGCTTG

all3759_fw	CGCTCGATGGTGACAAATGC

all3759_rv	TCCGAAATATCTTGACTTCCAAGAG

**translational fusion with the *gfp* gene**

2283 Prom-gfp_Fw	AATGCGTGGAGACCGCTGGTGCTTTCTTTAAAAATGACAA

2283 Prom-gfp_Rv	AAGTTCTTCTCCTTTCATATGTCTCCCGATAAATAGAATT

**Expression plasmids**

2283oe_Fw	GTTAGGAGAACCATATGTATTCCAATGTAAACACCAAGATT

2283oe_Rv	TCGAGGATCCACATATGTTAATACTGTGTAACTGTTTGCA

all2283-NdeI_fw	CGCGCGGCAGCCATATGTATTCCAATGTAAACACCAAGATT

all2283-EcoRI_rv	GACGGAGCTCGAATTCTTAATACTGTGTAACTGTTTGCA

al2283 mut fw	TATGGATGCCTCTGGATTTGGCACTCTAC

al2283 mut rv	CCAGAGGCATCCATAAAAGTAACAAATTGAAAA

all2284-BamHI-fw	CAGGATCCGAATTGGAGTCACCGCACAGATTCAC

all2284-PstI-rv	CCTGCAGGCGCGCCGTCAGTCTCTTGCATTTGGTAGTTC

al2284 mut fw	CTTTACAGCTGCAGTCCGTCATGCTCA

al2284 mut rv	ACTGCAGCTGTAAAGCCCTCAATTAATGCAGT

(pCDF)sigC_Fw2	ATAAGAAGGAGATATACATATGCCAGCAACATCTTTTTACGC

(3HA)sigC_Rv	TGCCGCCGCCACTCAGAGATTCCAAATAGTCACGG

(sigC)3HA_Fw	ATCTCTGAGTGGCGGCGGCAGTTATCCG

(pCDF)sigE_Fw2	ATAAGAAGGAGATATACATATGTACCAAACAAAGCATGAATCCC

(3HA)sigE_Rv	TGCCGCCGCCAATCAACCAACCCTTAAGACG

(sigE)3HA_Fw	TTGGTTGATTGGCGGCGGCAGTTATCCG

(pCDF)3HA_Rv	GGTTTCTTTACCAGACTCGACTCGAGTTAGCCCGCGTAATCAGG

**Bacterial two hybrid**

all2284 dh Fw	TTTTCTGCAGGATGTCACCGCACACAGATTCA

all2284 dh RV	TTTTTGAATTCGAGTCTCTTGCATTTGGTAGTTCAACT

SigE dh Fw T18	TTTTCTGCAGGATGTACCAAACAAAGCATGAATCC

SigE dh Rv sigC T18-T25	TTTTTGAATTCGAAATCAACCAACCCTTAAGACGTT

SigC dh Fw T18	TTTTCTGCAGGATGCCAGCAACATCTTTTTACGC

SigC dh Rv sigC-T25/T18	TTTTTGAATTCGAACTCAGAGATTCCAAATAGTCACG

SigC dh Fw T25-SigC	TTTTCTGCAGGGATGCCAGCAACATCTTTTTACGC

SigC dh Rv T25-SigC	TTTTTGAATTCCTAACTCAGAGATTCCAAATAGTCAC

**RTqPCR**

RTall2283_Fw	ACACCAAGATTATTCAACCTAGTGG

RTall2283_Rv	ACTGTTTGCATAAAAGCTGCTTG

RT-nifH-Fw	CTGCTAACAACATCGCTCGC

RT-nifH-Rv	AGCTAATGCGCGGTACTCTT

RT-fdxH-Fw	TGGCTAGCTACCAAGTTAGATTGA

RT-fdxH-Rv	GTGCAGTTGGAACGAGGGTA

RT-nifB-Fw	TGACACCACCAGTTACAGGC

RT-nifB-Rv	CACTAACTACGCCAGGACGG

HetP F	TGGCTGGTAAATACTCTTGGG

HetP R	ACCTACTACTTCCAGATAGGC

hetR RT fw	GCGTCGTCTGCTTTACTCTG

hetR RT rv	CCCAGTCTTTCATCATGCGG

**References**

1. Xu, X., Rachedi, R., Foglino, M., Talla, E., and Latifi, A. (2022) Interaction network among factors involved in heterocyst-patterning in cyanobacteria. *Mol Genet Genomics* **297**: 999–1015 https://link.springer.com/10.1007/s00438-022-01902-5

2. Karimova, G., Pidoux, J., Ullmann, A., and Ladant, D. (1998) A bacterial two-hybrid system based on a reconstituted signal transduction pathway. *Proc Natl Acad Sci USA* **95**: 5752–5756 https://pnas.org/doi/full/10.1073/pnas.95.10.5752. Accessed March 19, 2025.

3. Bishe B, Taton A, Golden JW. 2019. Modification of RSF1010-Based Broad-Host-Range Plasmids for Improved Conjugation and Cyanobacterial Bioprospecting. iScience 20:216-228.

# 4. [Rachedi](https://pubmed.ncbi.nlm.nih.gov/?term=Rachedi+R&cauthor_id=36756754) R, [Risoul](https://pubmed.ncbi.nlm.nih.gov/?term=Risoul+V&cauthor_id=36756754) V , [Scholivet](https://pubmed.ncbi.nlm.nih.gov/?term=Scholivet+A&cauthor_id=36756754) A, [Foglino](https://pubmed.ncbi.nlm.nih.gov/?term=Foglino+M&cauthor_id=36756754) M , Latifi A. 2023. Evidence that the PatB (CnfR) factor acts as a direct transcriptional regulator to control heterocyst development and function in the cyanobacterium Anabaena PCC 7120. Mol Microbiol 119(4):492-504.

5. Menestreau M, Rachedi R, Risoul V, Foglino M, Latifi A. 2022. The CcdB toxin is an efficient selective marker for CRISPR-plasmids developed for genome editing in cyanobacteria. MicroPubl Biol 2022.

**Figure S1 : Full-field micrographs corresponding to Fig. 1C.**

Microscopic bright field images (left), auto-fluorescence images (middle), and GFP fluorescence (right) of the indicated *Anabaena* strains grown in BG11 (top) or 24 hours after nitrogen stepdown (bottom). White boxes indicate the regions shown in the main figure.

**Figure S2: Alignment of NfsS (A) and NfsR (B) with homologous sequences.**

The kinase NfsS (**A**) and the anti-anti-sigma NfsR (**B**) from *Anabaena* were aligned with selected homologs using Clustal Omega. Catalytic residue N56 in NfsS and the predicted phosphoacceptor site S51 in NfsR are marked in red. Conserved residues are indicated by asterisks. Sequence names are listed to the left.

**Figure S3: Purification of His-NfsR and His-NfsS, and production of SigC-HA and SigE-HA.**

**A.** SDS-PAGE analysis of the indicated purified His₆-tagged NfsS and NfsR proteins, including wild-type proteins and the indicated variants. One microgram of each protein was loaded.

**C.** Immunoblot analysis of SigC-HA and SigE-HA production in *E. coli* using an anti-HA antibody. I, induced condition; NI, noninduced condition; lanes 1 and 2, independent biological replicates.

**D.** SDS-PAGE analysis of SigC-HA or SigE-HA produced in the presence (+) or absence (−) of His₆-NfsS. I, induced condition; NI, noninduced condition. Red asterisks indicate SigC-HA or SigE-HA, and red hash marks indicate His₆-NfsS.

**Figure S4: Uncropped immunoblot corresponding to Fig. 3B.a. Purification of SigC-HA co-produced with His-NfsS.**

Immunoblot analysis of the nickel-affinity co-purification of His₆-tagged NfsS coexpressed with 3xHA-tagged SigC in *E. coli*. M, molecular mass marker (kDa); L, loaded cell lysate; F, flow-through; W, wash fraction; 1–6, elution fractions. Black boxes indicate the regions shown in the main figure.

**Figure S5: Uncropped immunoblot corresponding to Fig. 3B.b. Purification of SigE-HA co-produced with His-NfsS.**

Immunoblot analysis of the nickel-affinity co-purification of His₆-tagged NfsS coexpressed with 3xHA-tagged SigE in *E. coli*. M, molecular mass marker (kDa); L, loaded cell lysate; F, flow-through; W, wash fraction; 1–6, elution fractions. Black boxes indicate the regions shown in the main figure.

**Figure S6: Uncropped immunoblot corresponding to Fig. 3.B.c Purification of His-NfsS produced in the absence of SigC-HA and SigE-HA.**

His-NfsS was produced and purified in the absence of SigC-HA or SigE-HA. Lanes 1 and 2 contain cell extracts corresponding to 0.01 and 0.001 OD units, respectively, of *E. coli* producing SigC-HA and were used as positive controls for anti-HA detection. Lanes 3–6 contain elution fractions corresponding to those in which SigC-HA and SigE-HA were detected in the pull-down assays shown in panels a and b. M, molecular mass marker (kDa). Black boxes indicate the regions shown in the main figure.

**Figure S7: Uncropped immunoblot corresponding to Fig. 3.B.d. Purification of SigC (top) or SigE (bottom) without NfsS**

Negative controls showing the nickel-affinity purification of HA-tagged SigC and SigE produced in the absence of His-NfsS, using the same procedure as in panels a and b. M, molecular mass marker (kDa); L, loaded cell lysate; F, flow-through; W, wash fraction; 1–6, elution fractions. Black boxes indicate the regions shown in the main figure.

**Figure S8**

**A : Characterization of the *nfsR* deletion mutant**

Left, genomic PCR analysis of the segregation of the Δ*nfsR* mutant. An internal of the *nfsR* gene was amplified. An internal fragment of all3759 was amplified as a positive control. The expected sizes of the PCR products are indicated below the gel. Right, growth of the indicated *Anabaena* strains in liquid BG11 or BG11₀ medium. Images were acquired 10 days after inoculation.

**B : Full view images for Fig.4B**

Bright-field images (top) and autofluorescence images (bottom) of the indicated Anabaena strains, including the ΔnfsR mutant, the mutant carrying a wild-type copy of nfsR, and the mutant producing the phosphoablative NfsR variant. Cells grown in BG11_0_ medium were imaged 24 h after combined-nitrogen step-down. White boxes indicate the regions shown in the main figure.
